# NOSIP overexpression promotes long-term persistence of CD8^+^ T cells during chronic infection

**DOI:** 10.3389/fimmu.2026.1755657

**Published:** 2026-06-22

**Authors:** Shihui Li, Toshikatsu Tamai, Yui Shinzawa, Sotaro Fujisawa, Yamato Tanabe, Makoto Utsunomiya, Hidetoshi Nakagawa, Junko Kurachi, Miki Koura, Eishiro Mizukoshi, Makoto Kurachi

**Affiliations:** 1 Department of Gastroenterology, Kanazawa University Hospital, Kanazawa, Japan; 2 Department of Molecular Genetics, Graduate School of Medical Science, Kanazawa University, Kanazawa, Japan; 3 Center for Biomedical Research and Education, Kanazawa University, Kanazawa, Japan; 4 Immune Network Research Unit, Pursuit of Truth Research Core, Institute for Frontier Science Initiative, Kanazawa University, Kanazawa, Japan; 5 Cancer Research Institute, Kanazawa University, Kanazawa, Japan; 6 Department of Gastroenterology, Kanazawa Medical University, Kanazawa, Japan

**Keywords:** CD8+ T cells, chronic antigen stimulation, CX3CR1 subset differentiation, NOSIP, PD-L1 blockade responsiveness

## Abstract

Chronic antigen exposure drives CD8^+^ T cell exhaustion; however, strategies to maintain a long-lived, functional CD8^+^ T cell population under chronic stimulation remain unclear. In this study, we demonstrate that overexpression of nitric oxide synthase–interacting protein (NOSIP) enhances the persistence of antigen-specific CD8^+^ T cells under chronic antigen stimulation. Notably, NOSIP overexpression preserved a less differentiated CX3CR1^neg^ subset and inhibited its progression toward an apoptosis-prone CX3CR1^hi^ state, which was associated with reducing cell death and promoting long-term persistence. In a tumor model, NOSIP-overexpressing CD8^+^ T cells exhibited improved tumor control, indicating that NOSIP-mediated persistence confers superior antitumor capacity. Furthermore, NOSIP overexpression increased the responsiveness of CD8^+^ T cells to programmed death-ligand 1 blockade, suggesting that NOSIP may represent a promising therapeutic target.

## 1 Introduction

During chronic viral infections and tumor progression, persistent antigen stimulation induces CD8^+^ T cell exhaustion, which is characterized by impaired effector functions, upregulation of immune checkpoint molecules, and increased apoptosis, ultimately resulting in inadequate long-term host protection (1, 2). Exhausted CD8^+^ T cells are heterogeneous and comprise two major subsets: progenitor-exhausted and terminally exhausted cells (3, 4). While progenitor-exhausted cells retain proliferative capacity and can respond to immunotherapies such as immune checkpoint blockades (ICBs), the dysfunctional state of terminally exhausted cells is epigenetically imprinted and irreversible. Consequently, ICB therapies, including programmed cell death protein 1 (PD-1)/programmed death-ligand 1 (PD-L1) and cytotoxic T-lymphocyte–associated protein 4 inhibitors, primarily act on the progenitor pool, leading to transient restoration of function (5–8). Although combination strategies—such as ICB with co-stimulatory agonists (e.g., 4-1BB, OX40) (9), cytokine support (e.g., IL-7) (10), and metabolic or epigenetic modulators (11, 12)—have been explored to improve ICB efficacy, maintaining durable CD8^+^ T cell responses under chronic antigen exposure remains challenging. This persistent limitation underscores the need to develop novel strategies that sustain long-lived, functional CD8^+^ T cells.

Previously, we longitudinally tracked peptide-specific CD8^+^ T cells from patients with hepatocellular carcinoma (HCC) who survived for more than 10 years following peptide vaccination (13). To investigate their long-term immune responses, we analyzed tetramer^+^ peripheral blood mononuclear cells (PBMC) collected five years after vaccination using single-cell RNA sequencing (scRNA-seq). Most peptide-specific CD8^+^ T cells were enriched in a dominant cluster with a memory-like phenotype and notably exhibited elevated expression of nitric oxide synthase–interacting protein (NOSIP), suggesting a potential association between NOSIP and durable T cell persistence.

NOSIP, a 34-kDa protein, was initially identified as a binding partner of endothelial nitric oxide synthase (eNOS) (14). It modulates the enzymatic activity of both eNOS and neuronal nitric oxide synthase (nNOS) by altering their subcellular localization, thereby attenuating nitric oxide (NO) production (15–17). NOSIP has been implicated in diverse processes, including vascular remodeling, mucosal secretion, neutrophil differentiation, gastrointestinal motility and astrogliogenesis (18–21). In addition to regulating NOS activity, NOSIP contains a U-box domain and functions as an E3 ubiquitin ligase that modulates protein phosphatase 2A (PP2A) signaling during brain and craniofacial development (22). It also participates in retinoic acid signaling by regulating Rbp1 expression, thereby influencing early neurogenesis (23). Despite these diverse biological roles, the function of NOSIP in lymphocytes—particularly in CD8^+^ T cells—remains poorly understood. Based on our clinical observation of elevated NOSIP expression in a memory-like CD8^+^ T cell cluster (13), we hypothesized that NOSIP may intrinsically modulate T cell persistence and function.

Here, we examined whether NOSIP overexpression could modulate CD8^+^ T cell differentiation and enhance their persistence in a chronic viral infection model. We found that NOSIP overexpression promotes the maintenance of a less differentiated CX3CR1^neg^ CD8^+^ T cell population and reduces apoptosis under chronic antigen stimulation. These findings identify a cell-intrinsic role for NOSIP in sustaining long-term CD8^+^ T cell responses, with potential implications for future combinatorial immunotherapy strategies.

## 2 Materials and methods

### 2.1 ScRNA-seq data processing

Datasets (13) under the bioproject number PRJDB11756 in the DNA data bank of Japan (DDBJ) used: HLA-A24:hTERT461 or HLA-A24:AFP357 tetramer^+^ PBMC from immunized HCC patients with tumor-associated antigen vaccines.

The sequenced reads were aligned to the GRCh38 reference transcriptome, and a filtered gene-barcode matrix was generated using CellRanger version 10.0.0. Seurat version 5.4.0 R toolkit (24) was used for quality control and downstream analysis of scRNA-seq data taking the gene-barcode matrix as input. Specifically, count table data were initially subjected to quality control measures, including the removal of low-quality cells and genes based on read counts (between 800 and 4000) and mitochondrial gene content (<10%). Normalization techniques were then applied to account for variations in sequencing depth and library size, followed by gene selection to filter out low-expressed genes. Subsequently, dimensionality reduction techniques such as principal component analysis (PCA) were employed to identify significant sources of variation, and graph-based clustering algorithm (Louvain algorithm) was utilized to group the single cells into 4 clusters using the top 17 principal components. Doublet removal was performed using DoubletFinder (25). Cell types (T cell, Monocyte, NK cell and B cell) were assigned to clusters through marker gene identification and annotation. CD8^+^ T cells within the T cell cluster were identified based on the following criteria: RNA count for CD8B >0 and CD4 ≤0. After the initial analysis, CD8^+^ T cells were re-clustered to discover two different subgroups (C0 and C1). The graph-based clustering algorithm (Louvain algorithm) was utilized to group the CD8^+^ T cells into subclusters with a resolution at 0.1. Differential expression analyses comparing gene expression in subgroups of CD8^+^ T cells were performed with the FindMarkers function of the Seurat package using the “poisson” method. Genes with P value <0.01 and log2 fold change >1 or <−1 were considered to be differentially expressed.

### 2.2 Mice

Wild-type (WT) C57BL/6 mice expressing CD45.1 were purchased from the Jackson Laboratory. These mice were crossed with CD45.2^+^ P14 mice transgenic for a TCR specific to the H-2Db gp33–41 epitope of Lymphocytic Choriomeningitis virus (LCMV) (provided by Dr. Ruka Setoguchi from RIKEN RCAI, Japan) to generate WT P14 mice expressing both CD45.1 and CD45.2, or CD45.1 alone. WT C57BL/6 mice expressing CD45.2 were purchased from Sankyo Labo Service Corporation, Inc. Male mice aged between 6 and 10 weeks were utilized for experiments. All animal experimentation was conducted in accordance with the guidelines approved by the Committee on Animal Experimentation of Kanazawa University (AP21-017-01, AP23-017-01).

### 2.3 Anesthesia, blood collection, and euthanasia

Mice were anesthetized with inhaled isoflurane delivered via an induction chamber connected to a vaporizer (3.5% isoflurane). For longitudinal monitoring of LCMV-infected mice, retro-orbital blood samples were collected once per week under isoflurane anesthesia. On the terminal day, blood was collected under anesthesia, followed by euthanasia via cervical dislocation under deep anesthesia, and tissues were harvested for downstream analyses.

In the B16-gp33 tumor model, mice were anesthetized with isoflurane for tumor measurements on days 10 and 13. On day 15, tumor size was measured under anesthesia, after which mice were euthanized via cervical dislocation under deep anesthesia. Tumors were then harvested and weighed.

### 2.4 Virus infection

LCMV Armstrong (Arm) and Clone 13 (Cl13) strains were kindly provided by Dr. E. John Wherry, University of Pennsylvania. Each viral strain was produced and titrated as described (26). Mice were infected with LCMV following established protocols. For infection, a dosage of 2×10^5^ plaque-forming units (PFUs) of LCMV Arm was administered intraperitoneally, while 4×10^6^ PFUs of LCMV Cl13 were administered intravenously.

### 2.5 Plasmid construction, retroviral transduction, and adoptive transfer of CD8^+^ T cells

The Gateway technology was employed in the construction of the plasmid vector, following established methodologies. The retroviral vectors MSCV (murine stem cell virus)-ccdB-VEX (violet-excited fluorescent protein) and empty vector controls (MSCV-VEX) were donated by Dr. Warren S. Pear, University of Pennsylvania. The cDNA encoding NOSIP (MR204220; OriGene) was cloned into the MSCV-ccdB-VEX plasmid to generate MSCV-NOSIP-VEX.

Retroviral (RV) particles were generated by co-transfecting HEK293T cells with MSCV-based RV expression plasmid and the pCL-Eco packaging plasmid using Lipofectamine 3000 transfection kit (Invitrogen) or jetOptimus DNA Transfection Reagent (PolyPlus). Prior to RV transduction, cell culture plates were coated with anti-mouse CD3ϵ (145-2C11; BioLegend) and anti-mouse CD28 (37.51; BioLegend) antibodies at concentrations of 5 μg/mL and 2 μg/mL, respectively, for a duration of 3 hours to facilitate T cell activation. Subsequently, bulk CD8^+^ T cells were isolated from the spleens of naïve WT P14 mice using the EasySepTM Mouse CD8^+^ T cell isolation Kit (STEMCELL Technologies), ensuring purity of the cell population. Isolated CD8^+^ T cells were then stimulated on the pre-coated plates with 100 U/mL recombinant human IL-2 (Peprotech) for 24 hours to induce activation. Following activation, the CD8^+^ T cells were transduced with RV in the presence of Polybrene (4 μg/mL; Sigma-Aldrich), employing spin infection at 2000g for 60 minutes at 30 °C.

For non-sorted studies (e.g., **Supplementary Figure 2**), P14 cells were cultured for 4 hours after retroviral transduction, and 1×10^5^ of CD45.1^+^ P14 T cells were intravenously transferred into CD45.2^+^ recipient mice infected with LCMV virus 24 hours before.

For sorted studies, transduced P14 cells were cultured for 24 hours and sorted using the FACSAria Fusion (BD Biosciences) to isolate VEX^+^ P14 cells, indicating successful transduction. For sorting, the RV-transduced P14 cells were stained with antibodies against CD45.2, CD45.1, and CD8a, and VEX^+^ P14 cells were purified. Sorted cells were transferred using two designs: For single-transfer experiments, 1×10^5^ empty or NOSIP-overexpressing VEX^+^ CD45.1^+^ P14 cells were transferred into individual CD45.2^+^ recipient mice. For co-transfer experiments, 1×10^5^ P14 cells were adoptively transferred at a 1:1 ratio of empty (CD45.1^+^CD45.2^+^) and NOSIP-overexpressing (CD45.1^+^) cells. In some experiments, the marker combination was reversed (i.e., empty CD45.1^+^ and NOSIP CD45.1^+^CD45.2^+^) to counterbalance any labeling bias. Recipient mice were infected with LCMV Cl13–48 hours prior to transfer.

### 2.6 Adoptive transfer of CD8^+^ T cells to uninfected mice

Prior to RV transduction, cell culture plates were coated with anti-mouse CD3ϵ and anti-mouse CD28 antibodies at concentrations of 1 μg/mL and 0.5 μg/mL, respectively, for 3 hours to facilitate T cell activation. Subsequently, bulk CD8^+^ T cells were isolated from the spleens of naïve WT P14 mice using the EasySepTM Mouse CD8^+^ T cell isolation Kit. Isolated CD8^+^ T cells were then stimulated on pre-coated plates with 20 U/mL recombinant human IL-2 for 24 hours to induce activation. Following activation, the CD8^+^ T cells were transduced with RV in the presence of Polybrene (4 μg/mL), employing spin infection at 2000g for 60 minutes at 30 °C. P14 cells were cultured for 1 day after retroviral transduction, and 4×10^5^ VEX^+^ P14 cells were adoptively transferred at a 1:1 ratio of empty (CD45.1^+^CD45.2^+^) and NOSIP-overexpressing (CD45.1^+^) cells. After 7 days of adoptive transfer, spleens were harvested and analyzed.

### 2.7 Flow cytometry

Lymphocytes from peripheral blood, spleen, and liver were analyzed. Spleens and liver tissues were collected and homogenized using a 70 µm cell strainer. Liver immune cells were further purified using Percoll gradient centrifugation. Red blood cells were lysed using the RBC lysis buffer. The antibodies used for flow cytometry are listed in **Supplementary Table 1**.

For cell surface staining, 2×10^6^ cells were seeded in a 96-well plate and first resuspended in PBS with Fixable Viability Stain 440UV at 4 °C for 30 minutes to exclude dead cells from analysis. After washing, cells were then incubated with anti-mouse CD16/CD32 antibody for 15 minutes at room temperature to minimize non-specific binding. Following Fc blocking, cells were incubated with antibodies in staining buffer (PBS containing 2% fetal bovine serum and EDTA) at 4 °C for 30 minutes. The antibodies used included CD8, CD45.1, CD45.2, CD44, CD62L, CD27, KLRG1, CX3CR1, Ly108, PD-1, TIGIT, TIM3. For intracellular cytokine staining, cells were incubated with 1 μg/mL gp33 peptide (MBL) in the presence of 0.1% brefeldin A and 0.1% monensin (both from eBioscience) at 37 °C for 4 hours. Following surface marker staining, cells were fixed with 2% PFA for 20 minutes at 4 °C to preserve VEX signal, followed by cytokine staining with Cytofix/Cytoperm Fixation/Permeabilization Kit (BD Biosciences). The antibodies used included: CD45.1, CD8, CD45.2, CD62L, CD44, TNF-α, granzyme B, IFN-γ, IL-2. For intranuclear transcription factor staining, cells were fixed with 2% PFA for 20 minutes at 4 °C to preserve VEX signal, fixed and permeabilized with the FoxP3/Transcription Factor Staining Buffer Set (eBioscience). The antibodies used included: CD45.2, CD62L, CD8a, CD45.1, PD-1, CD44, IRF4, EOMES, T-bet, Ki-67, TOX, TCF1/TCF7. Flow cytometry analysis was conducted using a FACSymphony A5 instrument, and data were analyzed using FlowJo v10 software (BD Biosciences).

### 2.8 Western blotting

Proteins were extracted from RV-transduced P14 CD8^+^ T cells that had been sorted for VEX^+^ expression 24 hours after transduction. Following sorting, a fraction of the cells was used for adoptive transfer, and the remaining cells were lysed for protein analysis. Cells were lysed in RIPA buffer (Millipore) supplemented with Protease Inhibitor Cocktail (Roche) and PhosSTOP (Roche). Protein concentration was determined using a BCA assay, and equal amounts of protein were loaded into SDS-PAGE gels with a 5–20% gradient (Fujifilm). The proteins were then transferred to PVDF membrane Immobilon-P (Millipore). Following transfer, membranes were blocked with 5% skim milk (Fujifilm) in TBST (Tris-buffered saline with Tween-20) for 1 hour and then incubated overnight at 4 °C with a primary rabbit polyclonal antibody against NOSIP (Abcam) diluted at 1:1000. β-actin served as an internal control (Cell Signaling Technologies). The next day, membranes were washed and incubated with HRP-linked anti-rabbit IgG secondary antibody (Cell Signaling Technologies) at room temperature for 30 minutes, followed by three additional washes with TBST. Finally, the protein bands were visualized using Clarity Western ECL Substrate kit (Bio-Rad).

### 2.9 Apoptosis analysis

CD8^+^ T cells were enriched on day 0 and retrovirally transduced on day 1 as described above. On day 2, transduced P14 cells were sorted to isolate VEX^+^ cells for downstream assays. Sorted VEX^+^ P14 cells were then co-cultured at a 1:1 ratio of empty (CD45.1^+^) and NOSIP-overexpressing (CD45.1^+^CD45.2^+^) cells (total 1×10^5^ cells per well) in 6-well plates under two stimulation conditions: (1) transient stimulation: cells were continuously stimulated by 100 U/mL recombinant human IL-2; (2) chronic stimulation: cells were continuous exposure to 1 ng/mL gp33 peptide and 10U/mL recombinant human IL-2. After 2 days of culture (day 4), cells were harvested and apoptosis was assessed using the MEBCYTO Apoptosis Kit (MBL). Cells were first incubated with anti-CD45.1, CD45.2, CX3CR1, and CD8 antibodies at 4 °C for 30 min in the dark, then washed twice with staining buffer. Cells were then resuspended in binding buffer and stained with annexin V-FITC (1:200) and Propidium Iodide (PI; 1:1000) at room temperature for 15 min in the dark. Stained cells were passed through a 70 µm nylon cell strainer and immediately analyzed by flow cytometry using the FACSymphony A5. Apoptotic status was defined as follows: live cells (Annexin V^−^ PI^−^), early apoptotic cells (Annexin V^+^ PI^−^), late apoptotic cells (Annexin V^+^ PI^+^), and necrotic cells (Annexin V^−^ PI^+^).

### 2.10 B16-gp33 tumor model

The right flank of WT C57BL/6 mice (recipient mice, CD45.2^+^) was shaved before subcutaneous injection of 2×10^5^ B16-gp33 cells at day 0. On day 3 after tumor cell inoculation, VEX^+^ P14 cells transduced with empty or NOSIP-overexpressing (2 × 10^5^ cells per mouse, CD45.1^+^) were sorted and adoptively transferred intravenously. Tumor dimensions (length and width) were measured on days 10, 13, and 15 using a digital caliper, and tumor volume was calculated using the formula: tumor volume = (4/3)×π×(length/2)×(width/2)². Mice were sacrificed on day 15, and the excised tumors were weighed to assess tumor weight.

### 2.11 Antibody treatment *in vivo*

PD-L1 blocking assays were performed following LCMV Cl13 infection and adoptive co-transfer of sorted VEX^+^ P14 cells as described above. Mice received intraperitoneal injections of 200 µg per dose per mouse of anti–PD-L1 antibody (clone 10F.9G2™; Bio X Cell) or rat IgG2b isotype control (LTF-2; Bio X Cell). Repeated injections of anti-PD-L1 or isotype control antibodies were administered starting on day 8 post-infection, then every 3 days for a total of 5 doses. All mice were sacrificed on day 23 post-infection for flow cytometry analysis.

### 2.12 NO assay

CD8^+^ T cells were enriched on day 0 and retrovirally transduced on day 1 as described above. On day 2, transduced P14 cells were sorted to isolate VEX^+^ cells for downstream assays. Sorted VEX^+^ P14 cells were then co-cultured at a 1:1 ratio of empty (CD45.1^+^) and NOSIP-overexpressing (CD45.1^+^CD45.2^+^) cells (total 1×10^5^ cells per well) in 6-well plates under chronic stimulation conditions (1 ng/mL gp33 peptide and 10 U/mL recombinant human IL-2). After 2 days of culture (day 4), cells were harvested, and intracellular NO was assessed using the Nitric Oxide Assay Kit (Flow Cytometry Orange; Abcam). Cells were first incubated with NO Orange Dye in 37 °C/5% CO_2_ incubator for 30 min, followed by a wash in PBS. As a positive control, cells were treated with NONOate in Assay Buffer at 37 °C. Then, cells were incubated with anti-CD45.1 and CD45.2 antibodies at 4 °C for 30 min in the dark, then washed twice with staining buffer. Stained cells were passed through a 70 µm nylon cell strainer and immediately analyzed by flow cytometry using the FACSCanto II (BD Biosciences).

### 2.13 Statistical analysis

All quantitative data are presented as mean ± SEM and were analyzed using GraphPad Prism software version 10.4.1. Paired t-tests and unpaired two-tailed Student’s t-tests with Welch’s correction were used for comparisons between two groups. For multiple group comparisons, either one-way repeated measures ANOVA with Holm–Sidak’s multiple comparisons test or two-way ANOVA with Šídák’s multiple comparisons test was applied, as appropriate. *P*-values < 0.05 were considered statistically significant. Statistical significance is indicated as follows: *P < 0.05, **P < 0.01, ***P < 0.001, ****P < 0.0001. Trends (0.05 < *P* < 0.1) are indicated where relevant.

## 3 Results

### 3.1 NOSIP is enriched in a memory-like CD8^+^ T cell cluster in peptide vaccine–treated patients

**Figure 1:**
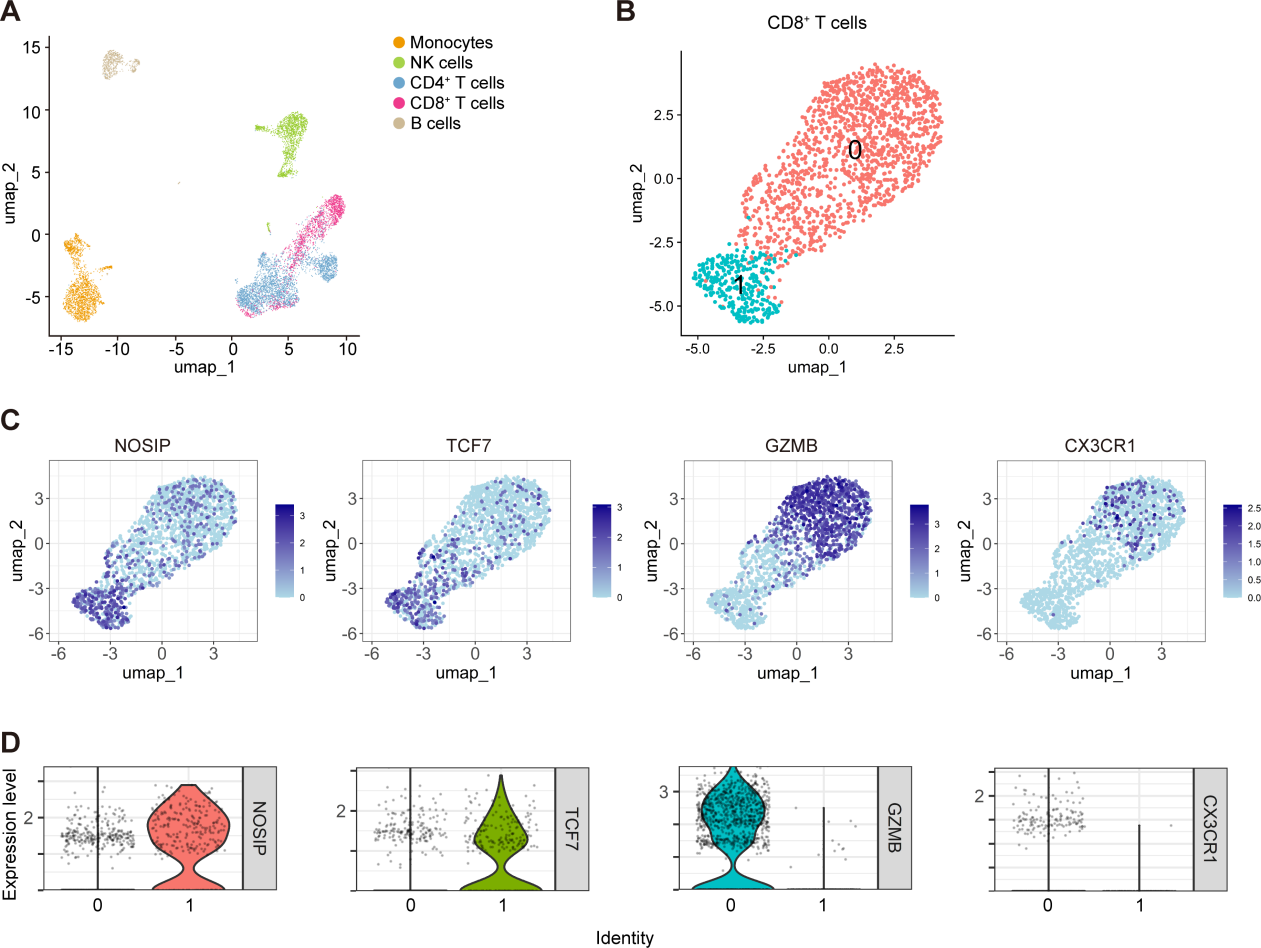
NOSIP expression in CD8^+^ T cell clusters identified by scRNA-seq analysis. **(A)** UMAP visualization of the combined scRNA-seq datasets from tetramer^+^ PBMC of peptide vaccine-treated HCC patients collected 1 and 5 years after vaccination. Unsupervised clustering identified major immune cell populations, including monocytes, NK cells, CD4^+^ T cells, CD8^+^ T cells, and B cells. **(B)** UMAP visualization of the CD8^+^ T cell subset extracted from the combined datasets shown in **(A)**. Unsupervised reclustering identified two CD8^+^ T cell clusters (cluster 0 and cluster 1). **(C)** Feature plots showing the expression patterns of representative genes across the CD8^+^ T cell clusters. NOSIP and TCF7 were preferentially expressed in cluster 1, whereas GZMB and CX3CR1 were enriched in cluster 0. **(D)** Violin plots showing the distribution of NOSIP, TCF7, GZMB, and CX3CR1 expression across the two CD8^+^ T cell clusters.

To further investigate the transcriptional context of NOSIP expression, we reanalyzed a previously reported scRNA-seq dataset of tetramer^+^ PBMC from peptide vaccine-treated HCC patients. PBMC collected at 1 and 5 years after vaccination were combined to enable an integrated analysis of the transcriptional landscape and were subjected to unsupervised clustering and UMAP visualization (**Figure 1A**). Unsupervised clustering of CD8^+^ T cells identified two major transcriptional clusters, visualized by UMAP projection (**Figure 1B**). Feature plot analysis revealed that NOSIP expression was preferentially enriched in cluster 1, which was characterized by high expression of the progenitor-associated transcription factor TCF7 (3), whereas cluster 0 exhibited elevated expression of the effector-associated genes GZMB and CX3CR1 (27, 28) (**Figure 1C**). Violin plot analysis further confirmed that NOSIP expression was higher in the TCF7^+^ CD8^+^ T cell cluster compared with the effector-like cluster (**Figure 1D**). Consistent with these observations, differential gene expression analysis demonstrated enrichment of NOSIP, TCF7, and SELL (29) in cluster 1, whereas GZMB and CX3CR1 were enriched in cluster 0 (**Supplementary Figure 1**). Together, these results place NOSIP within the transcriptional landscape of CD8^+^ T cell differentiation and indicate that NOSIP expression is associated with a memory-like CD8^+^ T cell cluster observed in long-term surviving patients following peptide vaccination.

### 3.2 NOSIP overexpression enhances CD8^+^ T cell persistence during chronic viral infection

Because NOSIP expression was enriched in a memory-like CD8^+^ T cell cluster identified by scRNA-seq analysis, we next asked whether NOSIP might influence CD8^+^ T cell differentiation and persistence *in vivo*. To evaluate the impact of NOSIP overexpression on these processes, we first performed single-transfer experiments using both the acute lymphocytic choriomeningitis virus (LCMV) Armstrong strain and the chronic LCMV Clone 13 (Cl13) strain—two well-established models for studying CD8^+^ T cell responses *in vivo* (30) (**Supplementary Figure 2A**). Equal numbers (1 × 10^5^) of CD45.1^+^ P14 cells (T cell receptor-transgenic cells specific for the LCMV gp33–41 epitope) transduced with retrovirus encoding empty or NOSIP were adoptively transferred into CD45.2^+^ recipient mice. Overexpression of NOSIP protein was confirmed by Western blot analysis (**Supplementary Figure 2B**). Following LCMV infection, empty and NOSIP-overexpressing P14 cells were analyzed within the VEX^+^ gate (**Supplementary Figure 2C**). In the acute infection model, the relative percentage of VEX^+^ P14 cells began to decline from day 15 in peripheral blood (PB), with NOSIP-overexpressing P14 cells exhibiting a slightly slower contraction than empty control (**Supplementary Figures 2D, E**). Notably, in the chronic infection model, NOSIP-overexpressing P14 cells tended to persist at higher frequencies in PB, particularly by days 29 and 42 (**Supplementary Figures 2F, G**). Consistently, NOSIP-overexpressing P14 cells remained at higher levels in the spleen on day 42 (**Supplementary Figures 2H–K**). These results indicate that NOSIP overexpression promotes the long-term persistence of CD8^+^ T cells, with a more pronounced effect under chronic antigen exposure.

**Figure 2:**
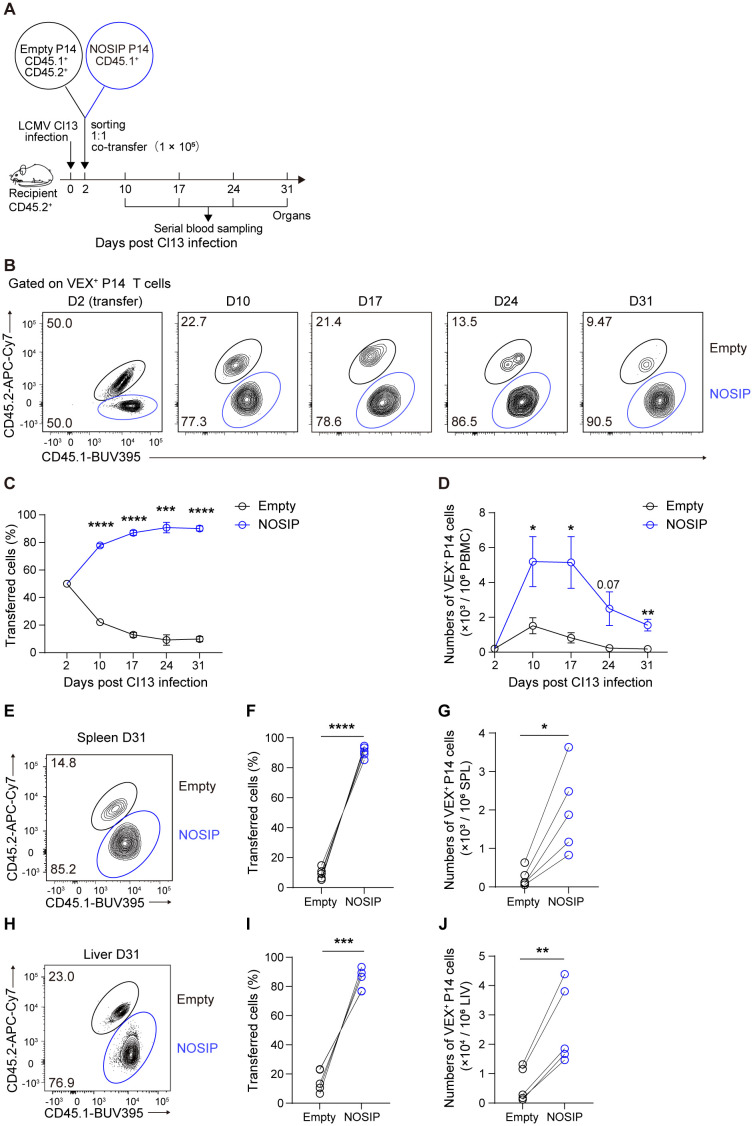
NOSIP enhances CD8^+^ T cell persistence during chronic viral infection. **(A)** Experimental scheme. CD45.2^+^ recipient mice were infected with LCMV clone 13 (Cl13) at day 0. On day 2, VEX^+^ empty and NOSIP-overexpressing P14 cells (CD45.1^+^ CD45.2^+^ and CD45.1^+^, respectively) were sorted, mixed at a 1:1 ratio and intravenously co-transferred into the recipient mice (1 × 10^5^ total cells per mouse). PB was collected at days 10, 17, 24, and 31, and spleen and liver were harvested at day 31. **(B)** Representative flow cytometry plots of transferred VEX^+^ P14 cells (gated on CD45.1^+^/CD45.2^+^) in PB at indicated time points. **(C, D)** Frequency **(C)** and quantification **(D)** of VEX^+^ P14 cells per 10^6^ PBMC over time (mean ± s.e.m., *n* = 5). **(E–G)** Persistence of transferred cells in spleen at day 31. Representative plots **(E)**, frequencies **(F)**, and numbers **(G)** of VEX^+^ P14 cells. **(H–J)** Persistence of transferred cells in liver at day 31. Representative plots **(H)**, frequencies **(I)**, and numbers **(J)** of VEX^+^ P14 cells. Cell counts were normalized to per million lymphocytes in each organ. Data are representative of two independent experiments. Data are presented as mean ± s.e.m. Statistical significance was determined using a paired two-tailed Student’s *t*-test. *P < 0.05; **P < 0.01; ***P < 0.001; ****P < 0.0001.

To strengthen these observations and enable direct comparison within the same host, we next performed co-transfer experiments using the Cl13 model. CD45.1^+^CD45.2^+^ or CD45.1^+^ P14 cells were retrovirally transduced with an empty or NOSIP encoding vector, respectively (**Figure 2A**). Equal numbers (5 × 10^4^) of VEX^+^ P14 cells were sorted on day 2 post-transduction and co-transferred into CD45.2^+^ recipient mice previously infected with LCMV Cl13 (day 0). The gating strategy for distinguishing empty and NOSIP-overexpressing P14 cells is shown in **Supplementary Figure 3**. Longitudinal flow cytometric analysis of PB revealed a progressive dominance of NOSIP-overexpressing P14 cells from day 10 to day 31 post-infection (**Figure 2B**). Although the initial input ratio was 1:1, a clear divergence appeared by day 10, with the ratio shifting to 1:9 by day 31 (**Figure 2C**). The numbers of VEX^+^ P14 cells in both groups peaked around day 10 post-infection. Between days 10 and 17, NOSIP-overexpressing P14 cells were stably maintained, whereas empty-transduced cells had already begun to decline. Empty P14 cells decreased sharply and were nearly lost by day 24, while NOSIP-overexpressing P14 cells contracted more gradually and persisted at significantly higher levels on day 31 (**Figure 2D**). To determine whether this effect extended beyond the circulation, we analyzed the distribution of VEX^+^ P14 cells in the spleen and liver on day 31. Consistent with the results observed in PB, NOSIP-overexpressing P14 cells exhibited significantly higher frequencies and absolute numbers in both tissues than empty control (**Figures 2E–J**). Collectively, these results demonstrate that NOSIP overexpression enhances the persistence of antigen-specific CD8^+^ T cells, particularly during chronic viral infection. In an additional transfer experiment using naïve, uninfected recipients, NOSIP-overexpressing P14 cells were also detected at a higher proportion on day 7 than empty control (**Supplementary Figure 4**), suggesting that NOSIP overexpression may confer a baseline persistence advantage after transfer. However, because the chronic infection model showed a clearer and more sustained divergence between the two populations, we focused our subsequent analyses on the chronic setting.

### 3.3 NOSIP overexpression sustains a less differentiated CX3CR1^neg^ CD8^+^ T cell population

**Figure 3:**
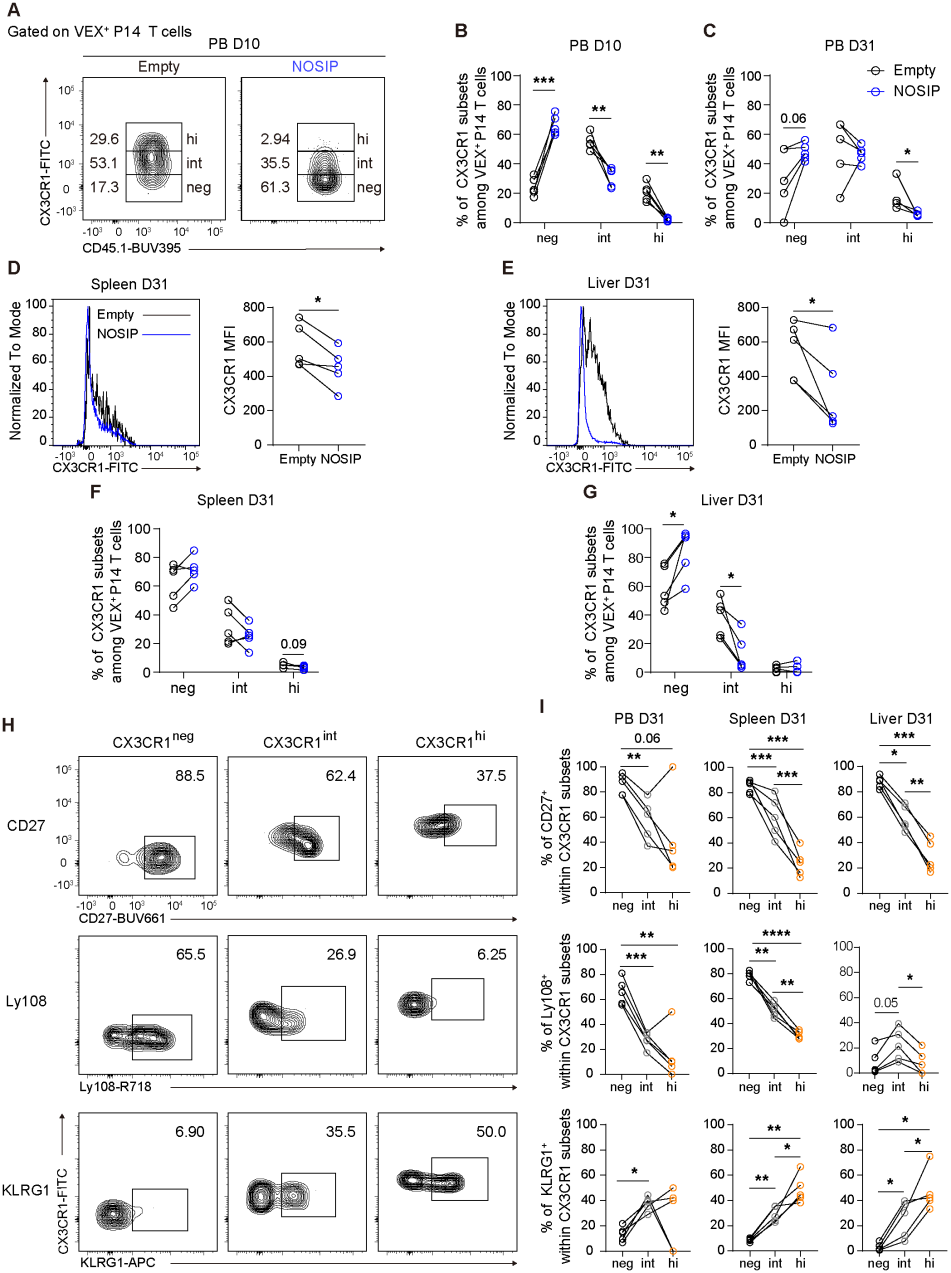
NOSIP overexpression promotes the maintenance of CX3CR1neg CD8^+^ T cells with less differentiated phenotypes. **(A)** Representative gating strategy for subsets defined by CX3CR1 expression (neg/int/hi) among VEX^+^ P14 cells in PB at day 10 post-infection. **(B, C)** Frequencies of CX3CR1^neg^, CX3CR1^int^, and CX3CR1^hi^ subsets among VEX^+^ P14 cells among empty and NOSIP-overexpressing groups in PB at day 10 **(B)** and day 31 **(C)**. **(D, E)** Representative histograms and quantification of CX3CR1 mean fluorescence intensity (MFI) in spleen **(D)** and liver **(E)** at day 31. **(F, G)** Frequencies of CX3CR1^neg^, CX3CR1^int^, and CX3CR1^hi^ subsets among VEX^+^ P14 cells in spleen **(F)** and liver **(G)** at day 31. **(H)** Representative plots showing CD27, Ly108, and KLRG1 expression within CX3CR1^neg^, CX3CR1^int^, and CX3CR1^hi^ subsets of NOSIP-overexpressing P14 cells. **(I)** Expression of CD27 (top), Ly108 (middle), and KLRG1 (bottom) within CX3CR1 subsets in PB, spleen, and liver at day 31. The data are representative of two independent experiments. Statistical significance was determined using a paired two-tailed Student’s t-test or one-way repeated measures ANOVA with Holm–Sidak’s multiple comparisons test. *P < 0.05; **P< 0.01; ***P< 0.001; ****P< 0. 0001.Trend (0.05 < *P* < 0.1) is indicated where relevant.

To further characterize the phenotype of NOSIP-overexpressing CD8^+^ T cells, we performed surface marker profiling to evaluate activation and memory (CD44, CD62L), differentiation (CD27, CX3CR1, Ly108, KLRG1), and exhaustion states (PD-1, TIGIT, TIM-3) (**Supplementary Figure 5A**) (31). In PB, compared with empty control, NOSIP-overexpressing P14 cells consistently exhibited lower frequencies of CX3CR1^+^ cells and higher frequencies of PD-1^+^ cells (**Supplementary Figure 5B**). This difference was further demonstrated by a progressive rightward shift in CX3CR1 intensity among empty control from day 10 to day 31, whereas NOSIP-overexpressing P14 cells maintained lower levels (**Supplementary Figure 5C**). Given the established relationship between CX3CR1 expression and T cell differentiation (32, 33), we subdivided VEX^+^ P14 cells into three CX3CR1-defined subsets: negative (neg), intermediate (int), and high (hi) (**Figure 3A**). In PB, NOSIP-overexpressing P14 cells consistently displayed higher frequencies of CX3CR1^neg^ cells and lower frequencies of CX3CR1^hi^ cells than empty control at both day 10 and day 31 post-infection (**Figures 3B, C**). Interestingly, cell number analysis showed that CX3CR1^neg^ cells were also increased in number, whereas CX3CR1^hi^ cells were not reduced in number and were even significantly increased on day 31, despite their lower frequency (**Supplementary Figure 5D**). A similar pattern was observed in the spleen and liver on day 31 (**Figures 3D, E**). Correspondingly, subset distributions shifted toward a greater proportion of CX3CR1^neg^ (**Figures 3F, G**). Moreover, in the single-transfer LCMV Cl13 model, longitudinal PB analysis from day 8 to day 42 revealed that NOSIP-overexpressing P14 cells maintained a larger CX3CR1^neg^ pool, whereas this subset gradually declined in empty control (**Supplementary Figures 6A, B**). A comparable trend was observed in the spleen on day 42 (**Supplementary Figures 6C, D**). Collectively, these findings suggest that the long-term persistence advantage conferred by NOSIP overexpression is accompanied by sustained restraint on the transition toward high CX3CR1 expression.

Since previous reports have shown that CX3CR1^neg^ cells exhibit higher PD-1 expression than CX3CR1^hi^ cells (34), we next compared PD-1 levels across the three subsets. As expected, PD-1 expression was highest in the CX3CR1^neg^ subset and lowest in CX3CR1^hi^ cells (**Supplementary Figure 7A**), suggesting that elevated PD-1 levels in the NOSIP group reflect an increased proportion of CX3CR1^neg^ cells. Because previous studies have used Ly108 together with CX3CR1 to further characterize CD8^+^ T cell differentiation status (35, 36), we next incorporated this marker combination into our analysis. In splenic VEX^+^ P14 cells on day 31, NOSIP-overexpressing cells showed an increased frequency of Ly108^+^CX3CR1^−^ cells, which are considered progenitor-exhausted cells (**Supplementary Figures 7B, C**). These findings suggest that NOSIP overexpression is associated with the preservation of a less-differentiated Ly108^+^CX3CR1^−^ population. To further assess the differentiation status of the three CX3CR1-defined subsets (37), we examined the expression of established markers—CD27, Ly108 and KLRG1 (**Figures 3H, I**). In NOSIP-overexpressing cells, the CX3CR1^neg^ subset was enriched for CD27^+^, Ly108^+^, and KLRG1^−^ cells, consistent with a less differentiated phenotype, whereas the CX3CR1^hi^ subset was predominantly CD27^−^, Ly108^−^, and KLRG1^+^, indicative of terminal differentiation. Similar trends were observed in empty control (**Supplementary Figures 7D, E**), reinforcing the association between CX3CR1 expression and differentiation state. Collectively, these results demonstrate that NOSIP overexpression delays the transition of CX3CR1^neg^ cells toward the CX3CR1^hi^ terminal phenotype, thereby preserving a less differentiated pool that may contribute to its persistence advantage.

### 3.4 NOSIP overexpression enhances the antitumor activity of CD8^+^ T cells

To further evaluate the functional capacity of NOSIP-overexpressing CD8^+^ T cells, we first analyzed key transcription factors associated with T cell fate. The expression levels of TCF1, TOX, and EOMES did not differ significantly between empty and NOSIP-overexpressing P14 cells (**Supplementary Figures 8A, B**). In contrast, Ki-67 expression showed a trend toward higher levels in NOSIP-overexpressing P14 cells, suggesting a potentially enhanced proliferative capacity within the less differentiated pool. Because NOSIP overexpression preferentially sustains the CX3CR1^neg^ pool—which has been reported to exhibit lower cytotoxicity than CX3CR1^hi^ cells (34)—we next assessed cytokine production. Upon ex vivo stimulation of splenic P14 cells on day 31 post-infection in the LCMV Cl13 model, NOSIP-overexpressing cells produced levels of IFN-γ, TNF-α, IL-2, and granzyme B comparable to those of empty control (**Supplementary Figure 8C**), indicating that NOSIP overexpression did not impair cytokine production at this stage.

**Figure 4:**
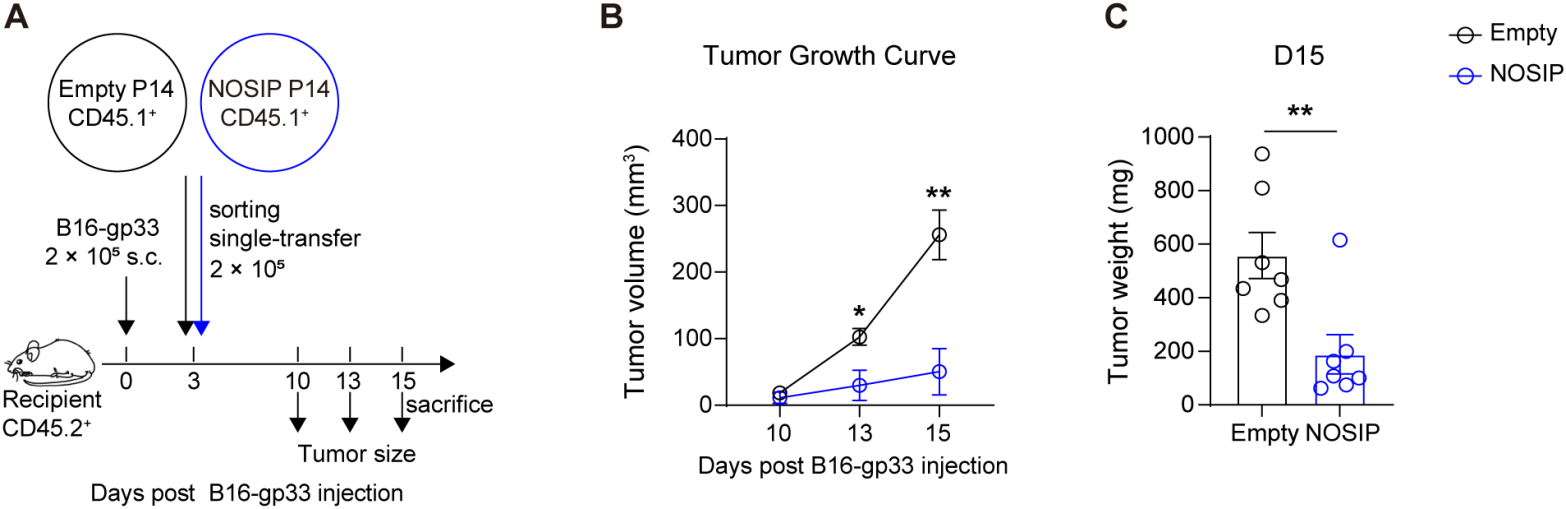
NOSIP overexpression suppresses tumor growth in the B16-gp33 tumor model. **(A)** Experimental scheme of the B16-gp33 tumor model. Recipient mice were subcutaneously injected with 2 × 10^5^ B16-gp33 cells (day 0). Sorted VEX^+^ CD45.1^+^ empty or NOSIP-overexpressing P14 cells (2 × 10^5^) were transferred on day 3. Tumor size was monitored on days 10,13 and 15. **(B)** Tumor growth curves showing tumor volume (mm³) at days 10, 13, and 15 post B16-gp33 injection. **(C)** Tumor weight at day 15 post B16-gp33 injection. The data are representative of two independent experiments. Data are presented as mean ± s.e.m. (n = 7 biological replicates). Statistical significance was determined using an unpaired two-tailed Student’s t-test. *P < 0.05; **P < 0.01.

Nevertheless, less differentiated CD8^+^ T cells have been shown to mediate superior tumor control *in vivo* compared with more differentiated counterparts (38). We next examined whether NOSIP-overexpressing cells could elicit effective antitumor responses *in vivo*. A B16-gp33 tumor model was employed, in which mice received adoptive transfers of either empty or NOSIP-overexpressing P14 cells (**Figure 4A**). Mice receiving NOSIP-overexpressing P14 cells exhibited significantly delayed tumor progression relative to empty control (**Figure 4B**). At the experimental endpoint, tumor weights were also significantly lower in the NOSIP-overexpressing group (**Figure 4C**), indicating that NOSIP-overexpressing CD8^+^ T cells retain the ability to mediate potent antitumor responses.

### 3.5 NOSIP overexpression enhances CD8^+^ T cell responsiveness to PD-L1 blockade

**Figure 5:**
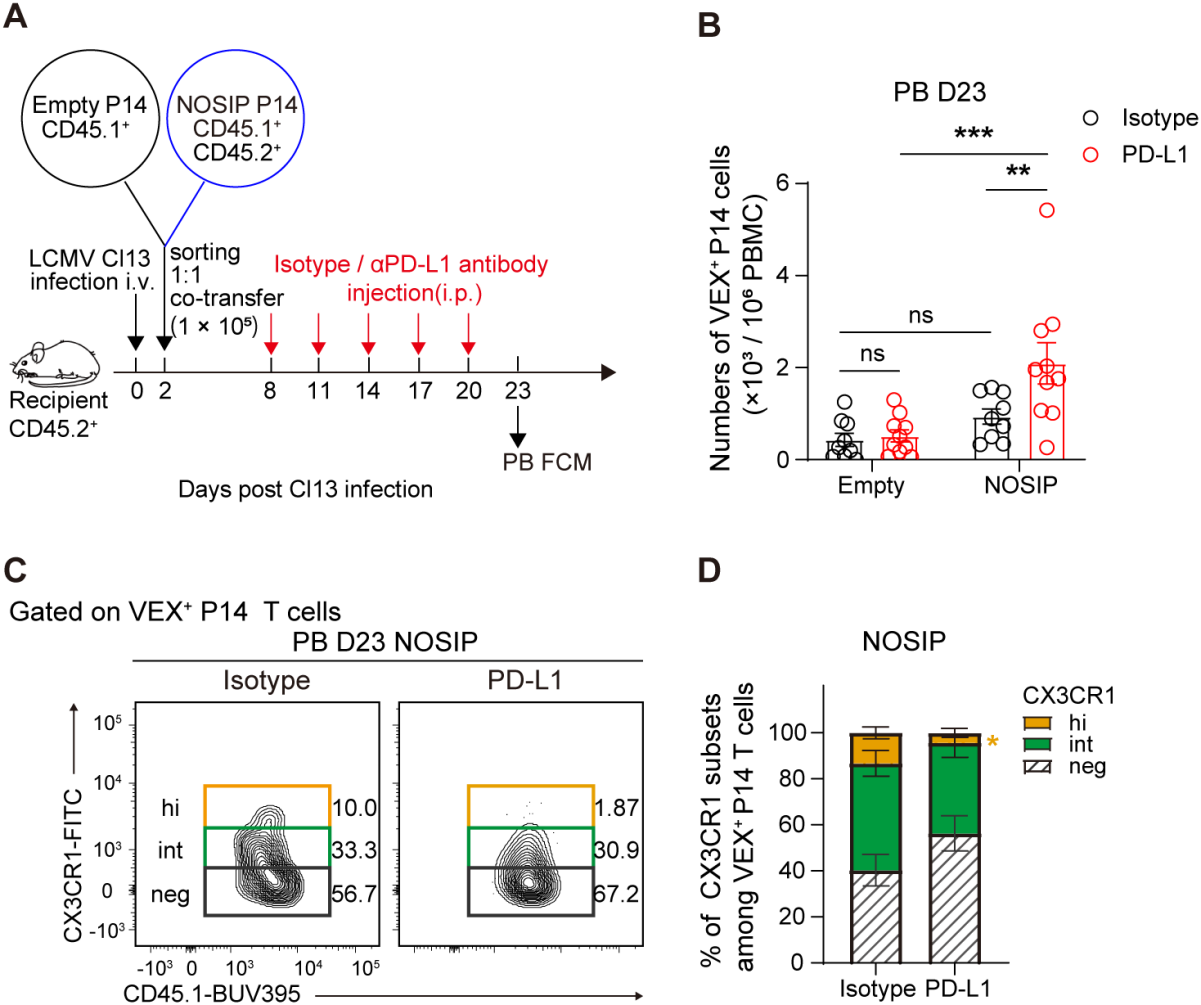
NOSIP overexpression enhances CD8^+^ T cell responsiveness to PD-L1 blockade. **(A)** Experimental scheme of anti–PD-L1 or isotype injection following co-transfer of empty and NOSIP-overexpressing P14 cells in the LCMV Cl13 infection model. **(B)** Quantification of VEX^+^ P14 cells per 10^6^ PBMC at day 23 post-infection. Two-way ANOVA indicated a trend toward a genotype × treatment interaction (p_interaction = 0.0518). **(C, D)** Representative flow cytometry plots **(C)** and quantification **(D)** of CX3CR1 subsets among NOSIP-overexpressing P14 cells in PB. * indicates a significant difference in the CX3CR1^hi^ subset between treatment groups. Data are pooled from two independent experiments. Bars represent mean ± s.e.m. Statistical significance was determined using two-way ANOVA with Šídák’s multiple comparisons test. *P < 0.05, **P < 0.01, ***P < 0.001. ns, not significant.

Given that ICB preferentially reinvigorates progenitor-like exhausted CD8^+^ T cells—a less differentiated subset with self-renewal capacity (4, 39)—we hypothesized that NOSIP overexpression would augment the proliferative expansion of CD8^+^ T cells in response to PD-L1 blockade. To test this hypothesis, LCMV Cl13-infected mice that had received co-transfers of empty and NOSIP-overexpressing P14 cells were treated with repeated administrations of anti-PD-L1 or isotype control antibodies (**Figure 5A**). By day 23 post-infection, NOSIP-overexpressing cells had become dominant in PB, increasing from 50% at transfer to approximately 75% in both isotype- and anti-PD-L1 antibody-treated groups (**Supplementary Figures 9A, B**). PD-L1 blockade significantly increased the numbers of NOSIP-overexpressing P14 cells while exerting minimal effects on the empty-transduced group (**Figure 5B**), suggesting that NOSIP overexpression enhances CD8^+^ T cell sensitivity to PD-L1 blockade.

Moreover, previous studies have demonstrated that progenitor-like populations preferentially expand following PD-L1 blockade (40). To determine whether a similar effect occurred in our system, we analyzed CX3CR1 expression in PB on day 23. Because of the limited number of empty P14 cells, subset analysis was restricted to the NOSIP-overexpressing P14 cells (**Figure 5C**). Anti-PD-L1 antibody treatment increased the CX3CR1^neg^ fraction while significantly reducing the proportion of CX3CR1^hi^ cells, indicating a shift toward a less differentiated phenotype (**Figure 5D**). Although the frequency of CX3CR1^neg^ cells showed only a modest upward trend, cell number analysis revealed a significant expansion of the CX3CR1^neg^ population, with CX3CR1^int^ cells also exhibiting an increasing trend and CX3CR1^hi^ cells maintained at comparable levels under anti-PD-L1 antibody treatment (**Supplementary Figure 9C**). Collectively, these results demonstrate that NOSIP overexpression enhances the proliferative response of CD8^+^ T cells to PD-L1 blockade and promotes the maintenance of a less differentiated CX3CR1^neg^ pool associated with long-term persistence.

### 3.6 NOSIP overexpression promotes CD8^+^ T cell persistence by limiting differentiation into an apoptosis-prone CX3CR1^hi^ state

**Figure 6:**
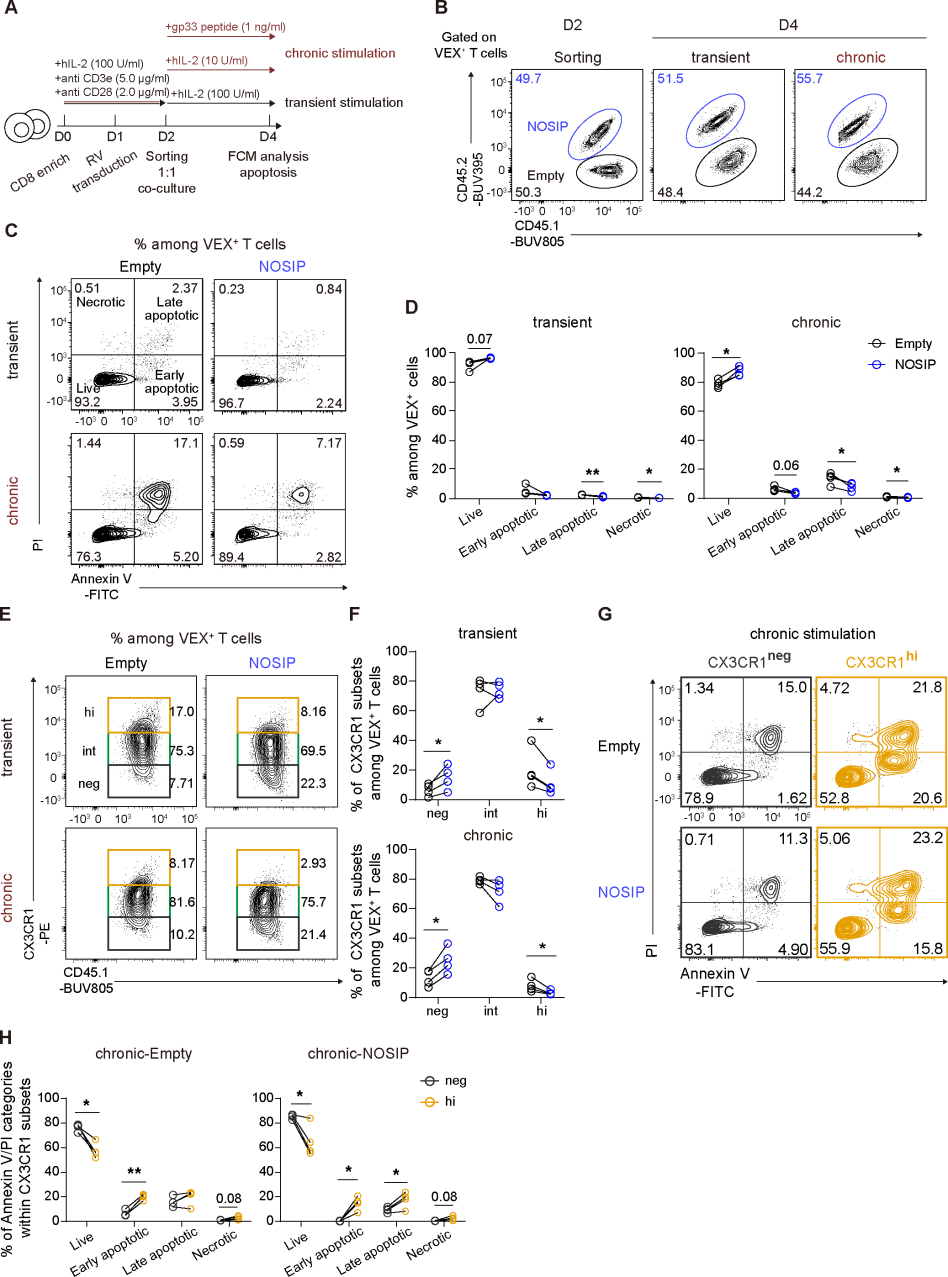
NOSIP overexpression reduces apoptosis of CD8^+^ T cells and preferentially maintains CX3CR1neg subsets *in vitro*. **(A)** Experimental scheme of the co-culture assay. Naive P14 cells were enriched on day 0, transduced with retrovirus encoding empty or NOSIP on day 1, sorted on day 2, and co-cultured at a 1:1 ratio under transient or chronic antigen stimulation. Cell apoptosis was assessed on day 4 by annexin V and PI staining. **(B)** Representative plots showing empty and NOSIP-overexpressing population. **(C)** Representative plots showing live (Annexin^−^ PI^−^), early apoptotic (Annexin^+^ PI^−^), late apoptotic (Annexin^+^ PI^+^), and necrotic (Annexin^−^ PI^+^) VEX^+^ P14 cells under transient or chronic antigen stimulation. **(D)** Quantification showing the percentage viability shown in **(C)**. **(E, F)** CX3CR1 subset distribution among VEX^+^ P14 cells comparing empty and NOSIP-overexpressing under transient and chronic stimulation, shown as representative plots **(E)** and subset frequencies (%) for CX3CR1^neg^, CX3CR1^int^, and CX3CR1^hi^ **(F)**. **(G, H)** Cell apoptosis determined by annexin V/PI within CX3CR1^neg^ and CX3CR1^hi^ subsets. **(G)** Representative plots showing annexin V and PI staining of CX3CR1^neg^ and CX3CR1^hi^ subsets in empty or NOSIP-overexpressing groups under chronic stimulation. **(H)** Annexin V/PI categories (%) (live, early apoptotic, late apoptotic, necrotic) within CX3CR1^neg^ and CX3CR1^hi^ subsets in empty or NOSIP-overexpressing groups under chronic stimulation. Data represent 4 biological replicates, and each experiment was performed with technical duplicates. Statistical significance was determined using a paired two-tailed Student’s t-test. *P < 0.05; **P < 0.01.

Because NOSIP-overexpressing CD8^+^ T cells exhibited enhanced antitumor activity and preferential maintenance of less differentiated phenotypes, we next sought to elucidate the mechanism by which NOSIP promotes CD8^+^ T cell persistence under chronic stimulation. Previous studies have demonstrated that less differentiated CD8^+^ T cell subsets possess greater self-renewal capacity and are less susceptible to apoptosis, whereas terminally differentiated cells are more prone to cell death (37). To investigate this, we performed *in vitro* co-cultures of equal numbers of sorted empty and NOSIP-overexpressing P14 cells under transient or chronic stimulation and analyzed their persistence (**Figure 6A**; **Supplementary Figure 10A**). The frequency of NOSIP-overexpressing P14 cells exhibited a modest increase compared with empty control under chronic stimulation on day 4, although the difference was not statistically significant (**Figure 6B**; **Supplementary Figure 10B**). Annexin V and PI staining revealed that NOSIP-overexpressing P14 cells consistently displayed lower frequencies of late apoptotic and necrotic cells, together with a higher proportion of live cells under chronic stimulation conditions (**Figures 6C, D**), with a similar but less pronounced trend observed under transient stimulation *in vitro*. These findings indicate that NOSIP overexpression is associated with reduced cell death, particularly under chronic stimulation.

Consistent with the findings from the LCMV infection model, NOSIP-overexpressing P14 cells maintained a higher proportion of CX3CR1^neg^ cells and a lower frequency of CX3CR1^hi^ cells *in vitro* under both stimulation conditions (**Figures 6E, F**). To determine whether the reduced cell death observed in NOSIP-overexpressing P14 cells was associated with the differentiation state, we next assessed annexin V/PI categories within CX3CR1 subsets under chronic stimulation (**Figure 6G**). The CX3CR1^hi^ subset exhibited significantly higher frequencies of apoptotic and necrotic cells, whereas the CX3CR1^neg^ subset was more resistant to cell death (**Figure 6H**). A similar pattern was observed under transient stimulation (**Supplementary Figures 10C, D**). Furthermore, intracellular cytokine staining demonstrated that NOSIP-overexpressing P14 cells produced levels of TNF-α, IFN-γ, and IL-2 comparable to those of empty control under both stimulation conditions (**Supplementary Figure 11**), consistent with the *in vivo* findings. Collectively, these results suggest that NOSIP overexpression is associated with enhanced CD8^+^ T cell persistence, accompanied by preservation of an apoptosis-resistant, less differentiated subset and reduced progression toward an apoptosis-prone terminal phenotype.

## 4 Discussion

Our study indicates that NOSIP overexpression prolongs CD8^+^ T cell persistence during chronic infection, accompanied by a restrained transition from a less differentiated CX3CR1^neg^ subset to a terminally differentiated CX3CR1^hi^ state. Consistent with this, NOSIP overexpression maintains a CX3CR1^neg^ pool that is more resistant to apoptosis. Notably, combining PD-L1 blockade with NOSIP overexpression resulted in a further enrichment of this persistent subset, suggesting that NOSIP may modulate differentiation trajectories to support sustained CD8^+^ T cell persistence. Interestingly, a recent scRNA-seq study of tumor-draining lymph nodes from patients with colorectal cancer reported that NOSIP expression progressively decreases along metallothionein-positive CD8^+^ T cell differentiation trajectories, further supporting our observation that NOSIP is associated with the maintenance of less-differentiated T cell states (41). To the best of our knowledge, our study provides the first functional evidence implicating NOSIP in regulating CD8+ T cell fate in a murine model of chronic infection, thereby extending its biological relevance to adaptive immunity.

Depletion of NOSIP has been reported to increase apoptosis in neural progenitor cells and neurons (23). NOSIP binds to NOS isoforms and suppresses their enzymatic activity, thereby reducing NO production. It may also function as an E3 ubiquitin ligase that inhibits PP2A signaling (16, 17). Both NO and PP2A pathways are well-established regulators of cell fate (42–44). A recent study demonstrated that NOSIP overexpression in hepatocellular carcinoma models suppressed apoptosis while increasing intracellular NO levels (45). Notably, NO has been shown to exert diverse effects and may not directly induce apoptosis in T cells (46), leaving the link between NOSIP-mediated NO/PP2A signaling and lymphocyte survival unresolved. In our model, NOSIP overexpression preferentially maintained the CX3CR1^neg^ subset, which exhibited significantly lower rates of apoptosis than the terminally differentiated CX3CR1^hi^ subset. This observation is consistent with previous findings that less differentiated CD8^+^ T cells are intrinsically more resistant to apoptosis and possess greater self-renewal capacity (34, 37). However, in the *in vitro* model, the differences between the empty control and NOSIP-overexpressing P14 cells were less pronounced than those observed *in vivo*. These findings suggest that, in addition to apoptosis resistance, multiple factors may contribute to the phenotype observed *in vivo*. Although NOSIP overexpression did not enhance cytokine production under chronic stimulation, this pattern aligns with the functional profile of progenitor-like cells, which primarily serve as a proliferative reservoir rather than immediate cytolytic effectors (4). The precise mechanism by which NOSIP restrains the progression from a less differentiated state to terminal differentiation remains to be elucidated. A recent study revealed that PP2A, as an interactor of the scaffold protein AMBRA1, participates in the differentiation program of CD8^+^ T cells through activation of transcription factor EB (47). Moreover, NO has also been reported to influence T cell development (48). In our preliminary analysis under chronic stimulation *in vitro*, NO Orange Dye staining showed a slight reduction in intracellular NO level in NOSIP-overexpressing P14 cells compared with empty control. However, the overall signal was minimal relative to the positive control, suggesting that only minimal NO production occurs under this condition (**Supplementary Figure 12**). Thus, while this result is consistent with the possibility that NOS-related signaling may be affected by NOSIP in this context, further studies will be required to clarify the underlying mechanism.

Although NOSIP has been primarily investigated in the context of NOS and PP2A regulation, emerging proteomic evidence suggests that its interaction network may be broader. For instance, a proteomic screen identified NOSIP as a potential cargo of the nuclear transport receptor importin 13 (16), implying that NOSIP may associate with molecular partners beyond the currently recognized NOS and PP2A, thereby expanding its functional repertoire. However, the NOSIP interactome in immune cells remains largely uncharacterized. Large-scale interactome and proteomic mapping platforms could be utilized to systematically identify NOSIP-binding partners and signaling networks in T cells (49, 50). Such analyses will be essential to elucidate how NOSIP modulates CD8^+^ T cell fate.

ICB exerts its therapeutic efficacy primarily through progenitor-like CD8^+^ T cells possessing self-renewal capacity (51–53). PD-L1 blockade has been reported to preferentially expand the CX3CR1^neg^ subset, whereas the CX3CR1^int^ and CX3CR1^hi^ subsets exhibit minimal changes (34). In our chronic infection model, PD-L1 inhibition markedly enhanced the expansion of NOSIP-overexpressing P14 cells in PB but had limited effects on empty control. Consistent with previous studies (4, 34), this expansion was accompanied by a phenotypic shift toward a less differentiated state, characterized by an increased frequency of CX3CR1^neg^ and a decreased frequency of CX3CR1^hi^ fractions. Importantly, this reduced frequency of CX3CR1^hi^ cells should not be interpreted as depletion of the effector-like compartment. In both the PD-L1 blockade setting and the co-transfer LCMV Cl13 infection model, the numbers of NOSIP-overexpressing CX3CR1^hi^ P14 cells were maintained at comparable levels or were even increased compared with empty control, whereas CX3CR1^neg^ cells were expanded, and CX3CR1^int^ cells showed a trend toward an increase. Thus, the observed phenotypic shift primarily reflects expansion of the less differentiated CX3CR1^neg^ population rather than loss of the CX3CR1^hi^ subset. These findings suggest that NOSIP maintains a progenitor-like CD8^+^ T cell compartment that remains preferentially responsive to PD-L1 blockade. Collectively, our results indicate that NOSIP not only sustains a persistent CD8^+^ T cell reservoir but also potentiates the therapeutic effects of immune checkpoint blockade.

While our study provides novel insights into the role of NOSIP in sustaining a less differentiated, apoptosis-resistant CD8^+^ T cell population, several limitations should be acknowledged. First, NOSIP has been reported to interact with eNOS and nNOS isoforms, which are primarily expressed in neuronal and vascular tissues (15, 18). However, its relevance to inducible nitric oxide synthase (iNOS)—the predominant isoform in immune cells (48, 54)—remains unclear. Whether NOSIP directly modulates iNOS activity in CD8^+^ T cells and how this interaction affects NO/PP2A signaling cascades requires further mechanistic investigation. Second, we did not directly assess the functional consequences of combining NOSIP overexpression with PD-L1 blockade, as the present study was specifically designed to delineate differentiation and persistence mechanisms. Future work using infection and tumor models will be necessary to address this limitation. Third, our study relied on NOSIP overexpression systems, which could induce nonphysiological alterations in cell differentiation. Therefore, we cannot conclude that NOSIP inherently contributes to the maintenance of less differentiated populations under physiological conditions. Definitive evidence will require NOSIP loss-of-function studies, such as knockout models. Finally, although our findings were established in a murine chronic infection model, their relevance to human immunity remains to be confirmed. Future studies employing human samples and clinically relevant tumor models will be essential to determine whether targeting NOSIP can preserve progenitor-like T cell reservoirs and enhance the durability of checkpoint-based immunotherapies. Collectively, these findings position NOSIP modulation as a promising strategy to sustain long-term T cell immunity in chronic infection and cancer.

## Data Availability

The raw data supporting the conclusions of this article will be made available by the authors, without undue reservation.
