# Correction: NOSIP overexpression promotes long-term persistence of CD8^+^ T cells during chronic infection

**DOI:** 10.3389/fimmu.2026.1933173

**Published:** 2026-07-21

**Authors:** Shihui Li, Toshikatsu Tamai, Yui Shinzawa, Sotaro Fujisawa, Yamato Tanabe, Makoto Utsunomiya, Hidetoshi Nakagawa, Junko Kurachi, Miki Koura, Eishiro Mizukoshi, Makoto Kurachi

**Affiliations:** 1 Department of Gastroenterology, Kanazawa University Hospital, Kanazawa, Japan; 2 Department of Molecular Genetics, Graduate School of Medical Science, Kanazawa University, Kanazawa, Japan; 3 Center for Biomedical Research and Education, Kanazawa University, Kanazawa, Japan; 4 Immune Network Research Unit, Pursuit of Truth Research Core, Institute for Frontier Science Initiative, Kanazawa University, Kanazawa, Japan; 5 Cancer Research Institute, Kanazawa University, Kanazawa, Japan; 6 Department of Gastroenterology, Kanazawa Medical University, Kanazawa, Japan

**Keywords:** CD8+ T cells, chronic antigen stimulation, CX3CR1 subset differentiation, NOSIP, PD-L1 blockade responsiveness

The funder “Japan Agency for Medical Research and Development, JP26fk0310531” to “Eishiro Mizukoshi” was erroneously omitted. The **Funding** statement has been revised to read:

“The author(s) declared that financial support was received for this work and/or its publication. This study was supported by Grant-in-Aid for Scientific Research (B: 23K24109 and 24K02435) and Grant-in-Aid for Research Activity Start-up (25K23807) from the Japan Society for the Promotion of Science, the Japan Agency for Medical Research and Development under Grant Number JP24fk0310509, JP25fk0310531, and JP26fk0310531, and the Kanazawa University SAKIGAKE project.”

The original version of this article has been updated.

