# Supplementary material for: NOSIP overexpression promotes long-term persistence of CD8+ T cells during chronic infection
Source: Front Immunol. 2026 Jun 22;17:1755657. doi: 10.3389/fimmu.2026.1755657 (PMC13333395; doi:10.3389/fimmu.2026.1755657)
Supplement: Supplementary file 1 [file DataSheet1.pdf]

Supplementary Material

1 Supplementary Table

Antigen	Fluorophore	Clone	Catalog No.	Company	Dilution
CD8a	BUV805	53-6.7	612898	BD	1/200
CD8a	SB702	53-6.7	67-0081-82	eBioscience	1/200
CD8a	BUV563	53-6.7	748535	BD	1/200
CD8a	PE	53-6.7	12-0081-83	eBioscience	1/200
CD45.1	BUV395	A20	565212	BD	1/200
CD45.1	BUV395	A20	363-0453-82	eBioscience	1/200
CD45.1	BUV805	A20	368-0453-82	eBioscience	1/200
CD45.1	Pacific Blue	A20	110722	eBioscience	1/200
CD45.2	PE-Cy7	104	109829	BioLegend	1/200
CD45.2	APC-Cy7	104	109824	BioLegend	1/200
CD45.2	APC-eF780	104	47-0454-82	eBioscience	1/200
CD45.2	BUV395	104	363-0454-82	eBioscience	1/200
CD45.2	BUV805	104	741957	BD	1/200
CD45.2	BUV737	104	612779	BD	1/200
TCRV α 2	APC	B20.1	17-5812-82	eBioscience	1/200
CD44	NB610	IM7	M010T02B06	invitrogen	1/200
CD44	APC-eF780	IM7	47-0441-80	eBioscience	1/200
CD62L	BUV563	MEL-14	741230	BD	1/200
CD62L	SB600	MEL-14	63-0621-82	eBioscience	1/200
CD27	BUV661	LG.3A10	741518	BD	1/200
CX3CR1	FITC	SA011F11	149020	BioLegend	1/200
CX3CR1	PE	SA011F11	149005	BioLegend	1/200
Ly108	R718	13G3	752059	BD	1/200
Ly108	BV786	13G3	741030	BD	1/200
KLRG1	APC	2F1	17-5893-80	eBioscience	1/200
PD-1	PE	RMP1-30	12-9981-81	eBioscience	1/200
PD-1	SB600	RMP1-30	63-9981-82	eBioscience	1/200
TIGIT	BV421	1G9	565270	BD	1/200
TIM3	SB702	RMT3-23	67-5870-82	eBioscience	1/200
IRF4	PerCP-eF710	3E4	46-9858-80	eBioscience	1/100
EOMES	PE-EF610	Dan11mag	61-4875-82	eBioscience	1/100
T-bet	PE-Cy5	4B10	15-5825-82	eBioscience	1/100
Ki-67	PE-Cy7	SolA15	25-5698-80	eBioscience	1/100
Ki-67	PerCP-Cy5.5	16A8	652423	BioLegend	1/100
TOX	APC	REA473	130-118-474	Miltenyi	1/100
TCF1/TCF7	PE	C63D9	14456	CST	1/100
Granzyme B	PE	NGZB	12-8898-80	eBioscience	1/100
TNF-α	BV750	MP6-XT22	506358	BioLegend	1/100
IL-2	APC	JES6-5H4	17-7021-81	eBioscience	1/100

IFN-γ	PE-Cy7	XMG1.2	25-7311-41	eBioscience	1/100

Supplementary Table 1. Antibody list for flow cytometry

2 Supplementary Figures

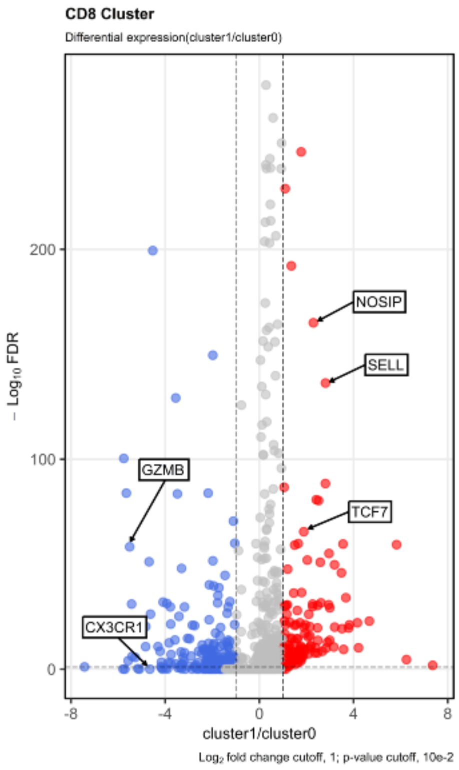

Supplementary Figure 1. Differential gene expression between CD8⁺ T cell clusters identified by scRNA-seq analysis

Volcano plot showing differentially expressed genes between cluster 1 and cluster 0 $CD8^+$ T cells. The x-axis represents the $\log_2$ fold change (cluster 1 vs cluster 0) and the y-axis represents the $-\log_{10}$ adjusted P value. Genes enriched in cluster 1 are shown in red, whereas genes enriched in cluster 0 are shown in blue. Selected genes, including NOSIP, SELL, TCF7, GZMB, and CX3CR1, are indicated.

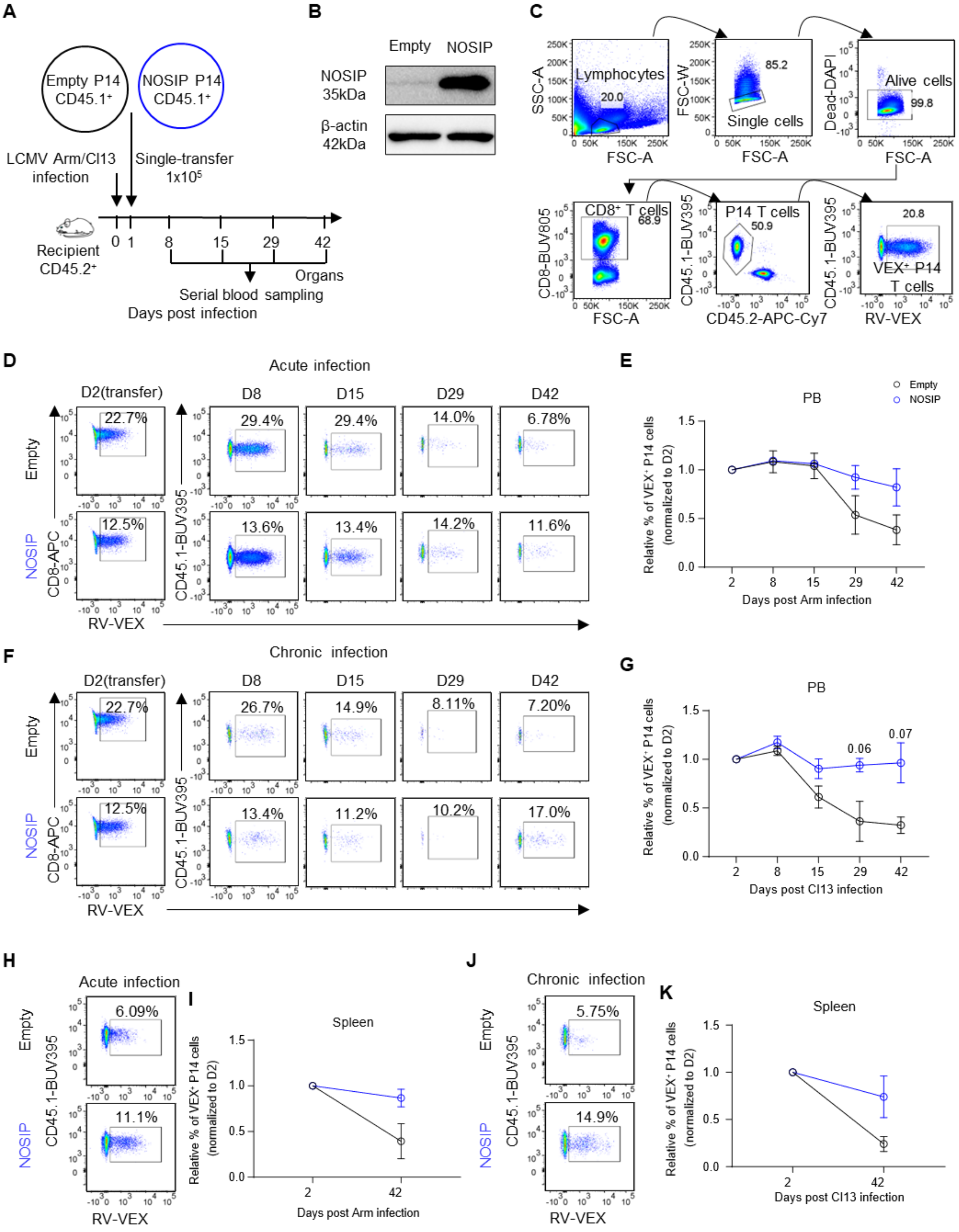
A
Empty P14 CD45.1+
NOSIP P14 CD45.1+
LCMV Arm/Cl13 infection
Single-transfer 1x10^5
Recipient CD45.2+
0 1 8 15 29 42
Organs
Serial blood sampling
Days post infection
B
Empty NOSIP
NOSIP 35kDa
β-actin 42kDa
C
Lymphocytes 20.0
Single cells 85.2
Alive cells 99.8
CD8+ T cells 68.9
P14 T cells 50.9
VEX+ P14 T cells 20.8
SSC-A
FSC-A
FSC-W
Dead-DAPI
CD8-BUV805
CD45.1-BUV395
CD45.2-APC-Cy7
RV-VEX
D
Acute infection
D2(transfer) D8 D15 D29 D42
Empty 22.7% 29.4% 29.4% 14.0% 6.78%
NOSIP 12.5% 13.6% 13.4% 14.2% 11.6%
CD8-APC
CD45.1-BUV395
RV-VEX
E
PB
Empty
NOSIP
Relative % of VEX+ P14 cells (normalized to D2)
Days post Arm infection
F
Chronic infection
D2(transfer) D8 D15 D29 D42
Empty 22.7% 26.7% 14.9% 8.11% 7.20%
NOSIP 12.5% 13.4% 11.2% 10.2% 17.0%
G
PB
0.06
0.07
Days post Cl13 infection
H
Acute infection
Empty 6.09%
NOSIP 11.1%
I
Spleen
Days post Arm infection
J
Chronic infection
Empty 5.75%
NOSIP 14.9%
K
Spleen
Days post Cl13 infection

Supplementary Figure 2. NOSIP enhances CD8$^+$ T cell persistence in both acute and chronic infection models under single-transfer, non-sorted conditions

(A) Experimental scheme. Recipient mice were infected with LCMV Armstrong (Arm) or Clone 13 (Cl13) at day 0 and received empty- or NOSIP-transduced P14 cells at day 1. Peripheral blood (PB) was collected at days 8, 15, 29, and 42, and spleens were harvested at day 42. **(B)** Western blot analysis of NOSIP expression in bulk (unsorted) P14 cells transduced with empty or NOSIP vectors. β-actin served as a loading control. **(C)** Flow cytometry gating strategy used to identify VEX$^+$ P14 cells in PB or spleen. **(D, E)** Acute infection model (LCMV Arm). Representative FACS plots of P14 cells in PB at the indicated time points (D), and longitudinal quantification of VEX$^+$ P14 cells in PB (E). **(F, G)** Chronic infection model (LCMV Cl13). Representative FACS plots of P14 cells in PB (F), and quantification of VEX$^+$ P14 cells over time (G). **(H–K)** Terminal spleen analysis (day 42). Representative spleen plots from LCMV Arms (H) and Cl13 (J). Quantification of VEX$^+$ P14 cells in spleen for Arm (I) and Cl13 (K). Data were normalized to day 2 due to differences in transduction efficiency. Values are presented as mean ± s.e.m. Statistical significance was assessed using unpaired two-tailed t-tests with Welch's correction to compare empty and NOSIP groups at individual time points. *$P < 0.05$; trends ($0.05 < P < 0.1$) are indicated where relevant. The experiment was performed once. The data shown are representative of this experiment.

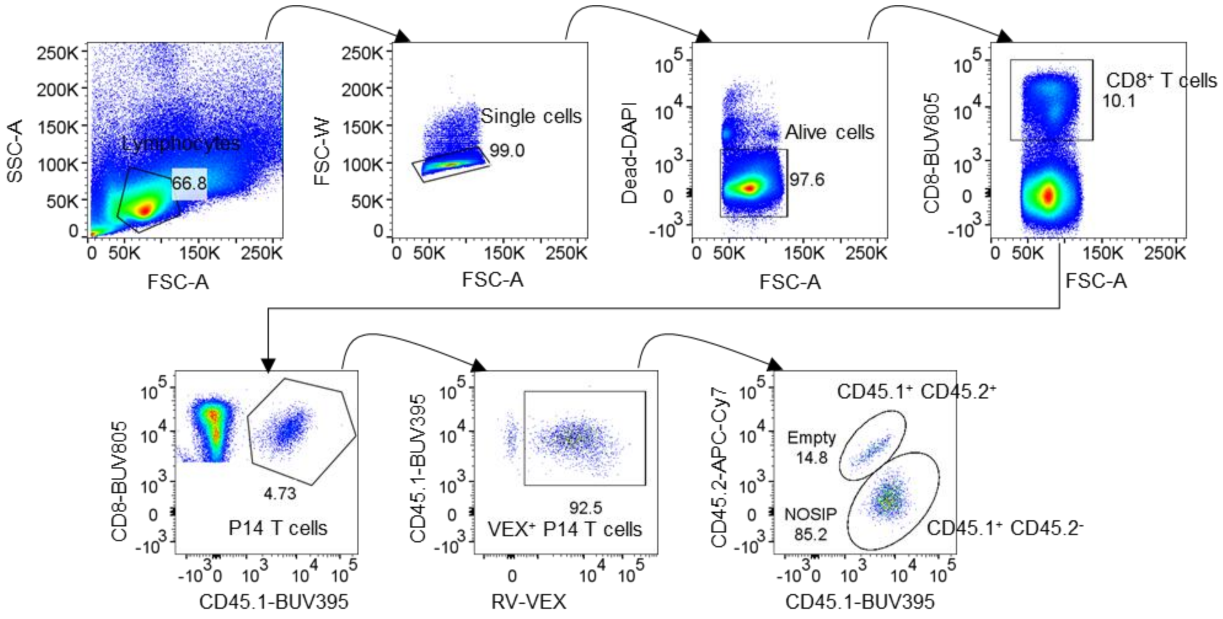

Supplementary Figure 3. Flow cytometry gating strategy

Representative flow cytometry gating strategies (spleen, day 31 post-infection) related to Figure 1(B-J). $CD45.1^+$ VEX^+ P14 cells were identified in wild-type recipients. Empty and NOSIP subsets were distinguished based on CD45.1/CD45.2 expression. Arrows indicate the gating workflow.

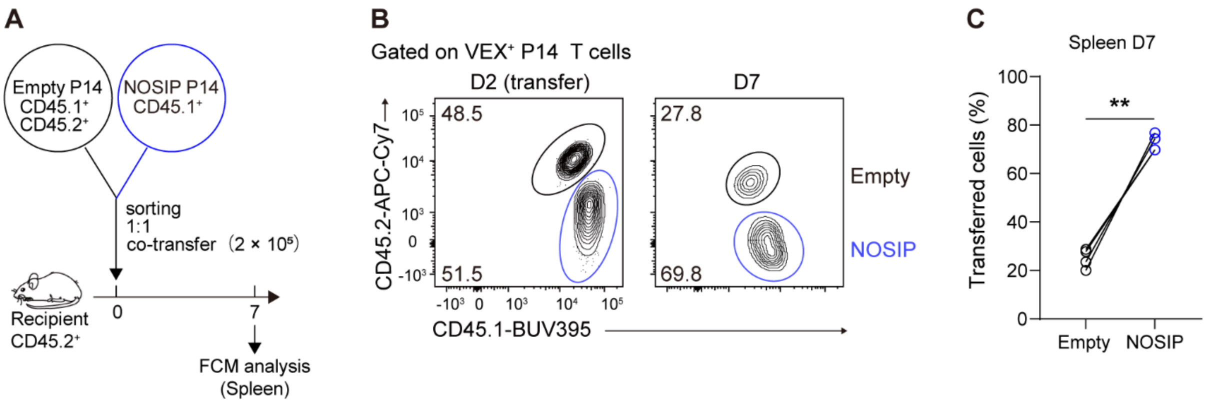

Supplementary Figure 4. NOSIP-overexpressing P14 cells are preferentially maintained after transfer into naïve recipients

(A) Experimental scheme. VEX^+ empty- and NOSIP-vector transduced P14 cells were mixed at an approximately 1:1 ratio and intravenously co-transferred into naïve recipient mice. Spleens were harvested at day 7 after transfer for flow cytometric analysis. (B) Representative flow cytometry plots showing the relative proportions of empty- and NOSIP-transduced cells among VEX^+ P14 cells at the time of transfer and in the spleen at day 7 after transfer. (C) Frequencies of transferred empty- and NOSIP-transduced cells among VEX^+ P14 cells in the spleen at day 7 (n=4). Statistical significance was determined using a paired two-tailed Student's t-test. $^{**}P < 0.01$.

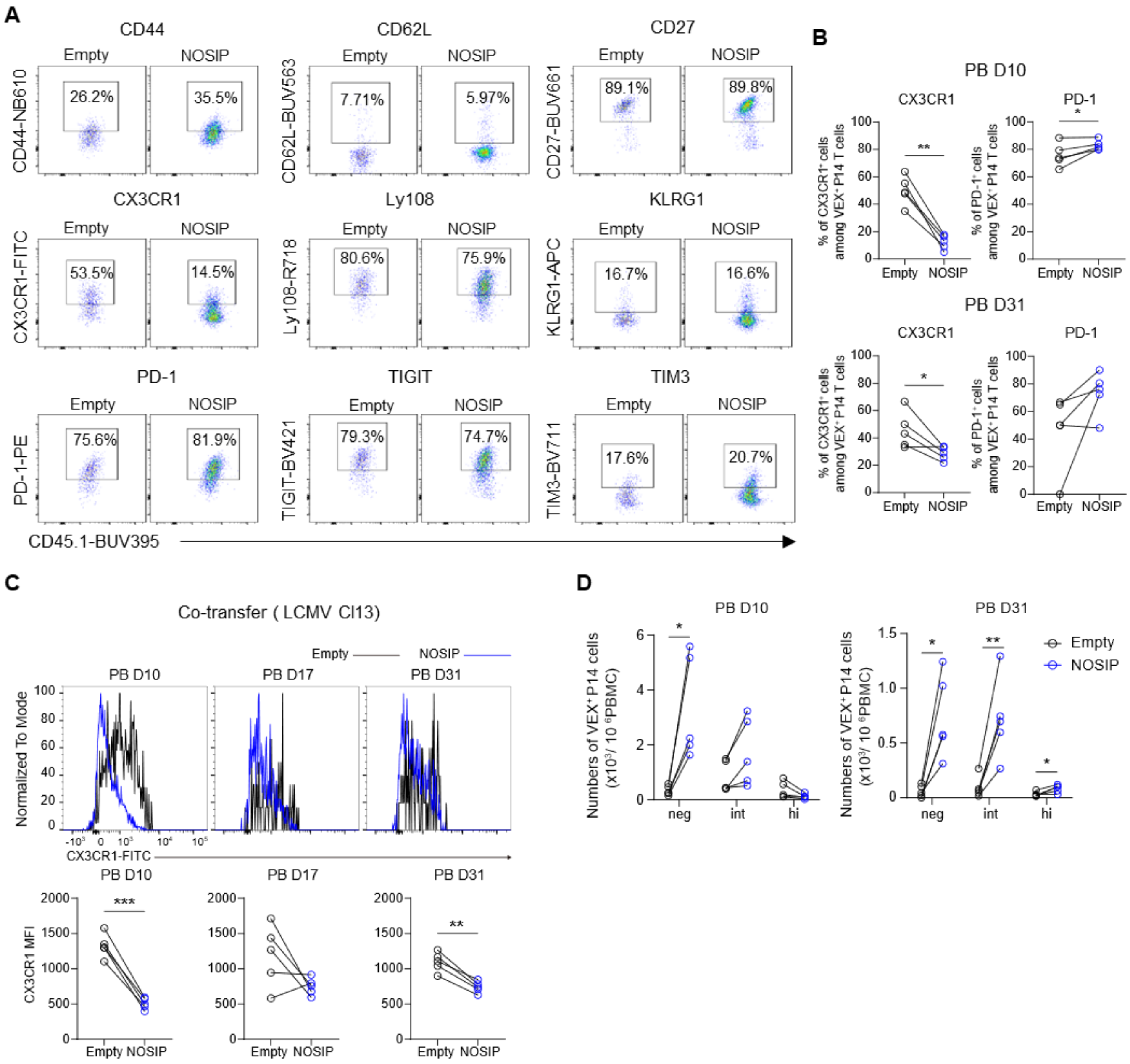

Supplementary Figure 5. NOSIP maintains CX3CR1^{neg} CD8^{+} T cell populations over time in the co-transfer setting

(A) Gating strategy for surface marker analysis of VEX^{+} P14 cells. Data shown are concatenated flow plots from empty- or NOSIP-transduced groups (PB, day 10), respectively. Assessed markers include CD44, CD62L, CD27, CX3CR1, Ly108, KLRG1, PD-1, TIGIT, and TIM3. **(B)** Frequencies of CX3CR1 and PD-1 expression among VEX^{+} P14 cells in PB at days 10 and 31. **(C)** Histograms (concatenated from all mice within each group) and quantification of CX3CR1 MFI over time (days 10, 17, and 31) in PB.

(D-G) Single-transfer in the LCMV Cl13 chronic infection model. **(D)** Quantification of VEX^+ P14 cells within the $CX3CR1^{neg}$, $CX3CR1^{int}$, and $CX3CR1^{hi}$ subsets per 10^6 PBMC in PB at days 10 and 31 post LCMV Cl13 infection. The data are representative of two independent experiments. Statistical significance was determined using a paired two-tailed Student's t-test. *$P < 0.05$; **$P < 0.01$; ***$P < 0.001$.

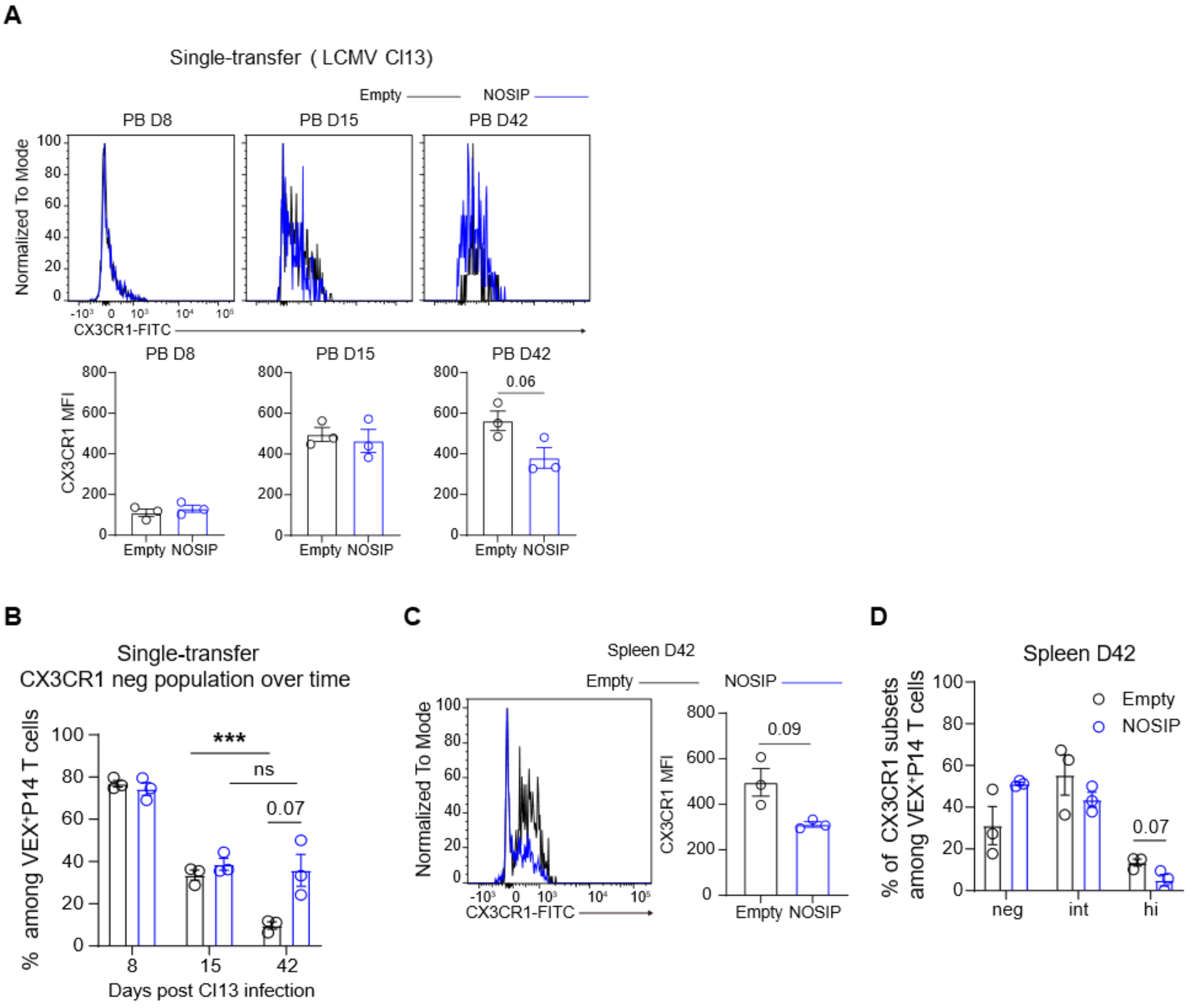

Supplementary Figure 6. NOSIP maintains CX3CR1^{neg} CD8^{+} T cell populations over time in the single-transfer setting

(A) Histograms (concatenated from all mice within each group) and quantification of CX3CR1 MFI over time (days 8, 15, and 42 post Cl13 infection) in PB. **(B)** Frequencies of CX3CR1^{neg} populations among VEX^{+} P14 cells in PB at days 8, 15, and 42. **(C)** Histograms (concatenated from all mice within each group) and quantification of CX3CR1 MFI in spleen at day 42. **(D)** Frequencies of CX3CR1 subsets in spleen at day 42. Data are presented as mean ± s.e.m. The single-transfer experiment was performed once. Statistical significance was assessed using unpaired two-tailed t-tests with Welch's correction. ns, not significant; ***$P < 0.001$. Trends ($0.05 < P < 0.1$) are indicated where relevant.

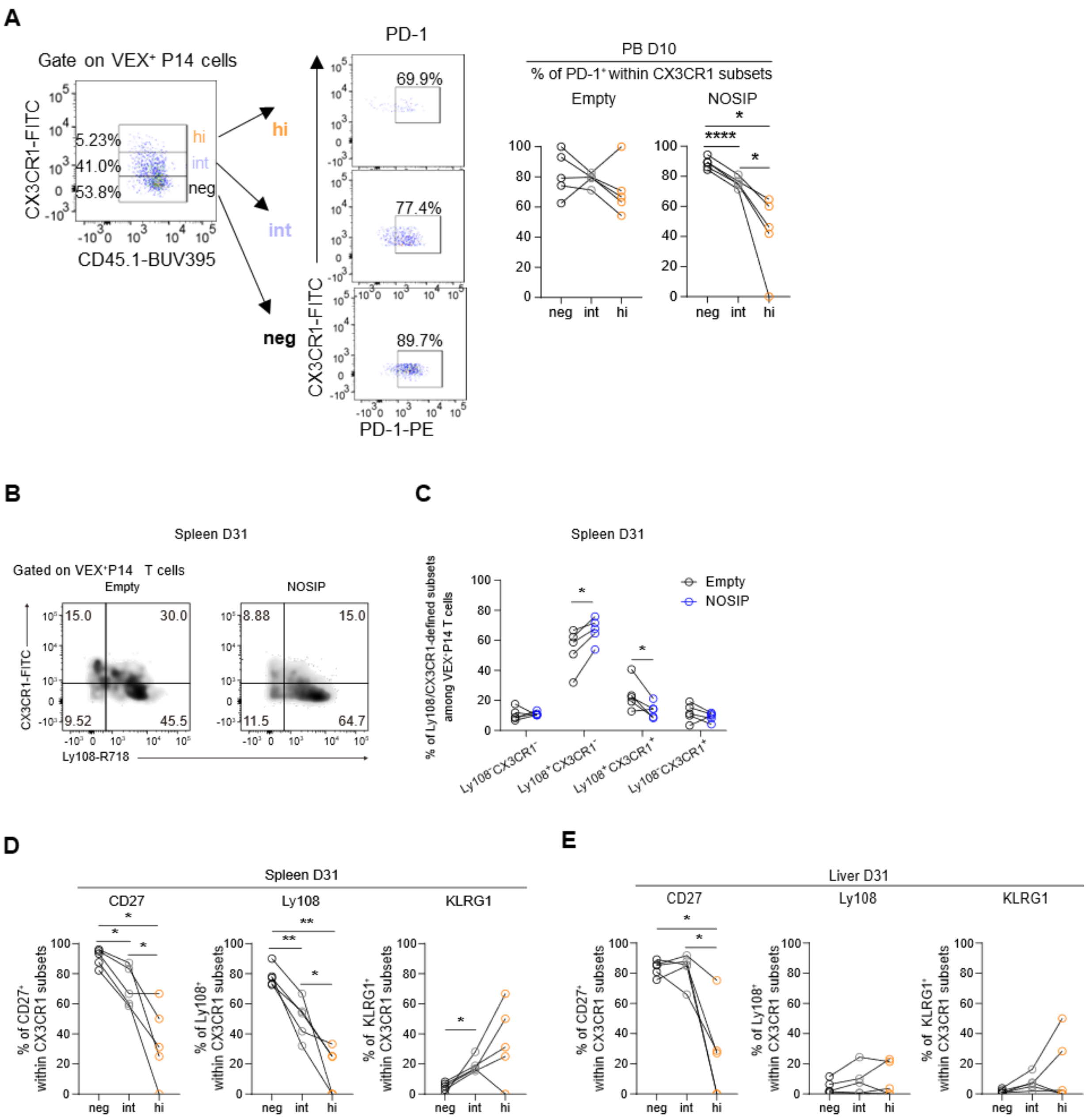

Supplementary Figure 7. PD-1 expression and differentiation-associated phenotyping of CX3CR1 subsets

(A) Gating strategy and comparison of PD-1 expression within CX3CR1^{neg}, CX3CR1^{int}, and CX3CR1^{hi} subsets at day 10 post Cl13 infection in PB. **(B, C)** Concatenated Ly108/CX3CR1 flow cytometry plots (B) and quantification of Ly108/CX3CR1-defined populations (C) among splenic VEX^{+} P14 cells at day 31 post Cl13 infection. **(D, E)** Frequencies of CD27, Ly108, and KLRG1 expression within CX3CR1 subsets in spleen (D) and liver (E) at day 31 post Cl13 infection in the empty group. The data are representative of two independent experiments. Statistical significance was determined

using one-way repeated measures ANOVA with Holm–Sidak's multiple comparisons test and a paired two-tailed Student's t-test. *$P < 0.05$; **$P < 0.01$, ****$P < 0.0001$.

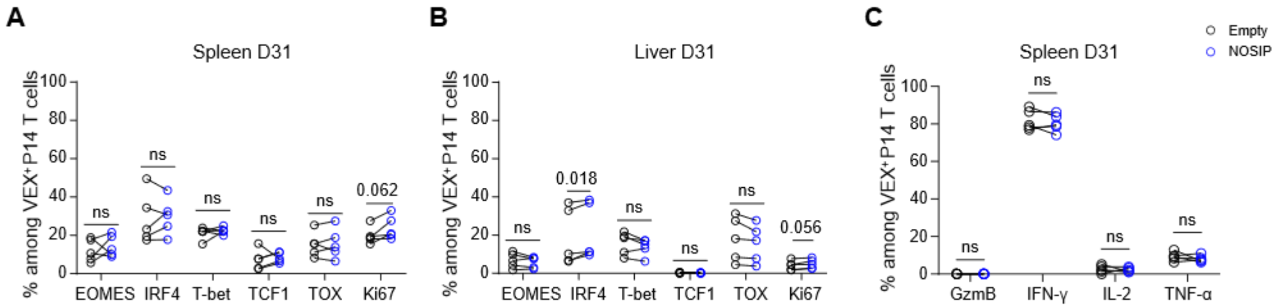

Supplementary Figure 8. Comparable transcription factor and cytokine expression between NOSIP- and Empty-transduced CD8$^+$ T cells

(A, B) Frequencies of transcription factors associated with memory and exhaustion states, including EOMES, IRF4, T-bet, TCF1, TOX, and the proliferation marker Ki-67, among VEX$^+$ P14 cells in spleen (A) and liver (B). **(C)** Frequencies of cytokine-producing (IFN-γ, IL-2, TNF-α) and cytotoxic (Granzyme B) VEX$^+$ P14 cells in spleen after ex vivo stimulation. The data are representative of two independent experiments. Statistical significance was determined using a paired two-tailed Student's t-test. ns, not significant. Trends ($0.05 < P < 0.1$) are indicated where relevant.

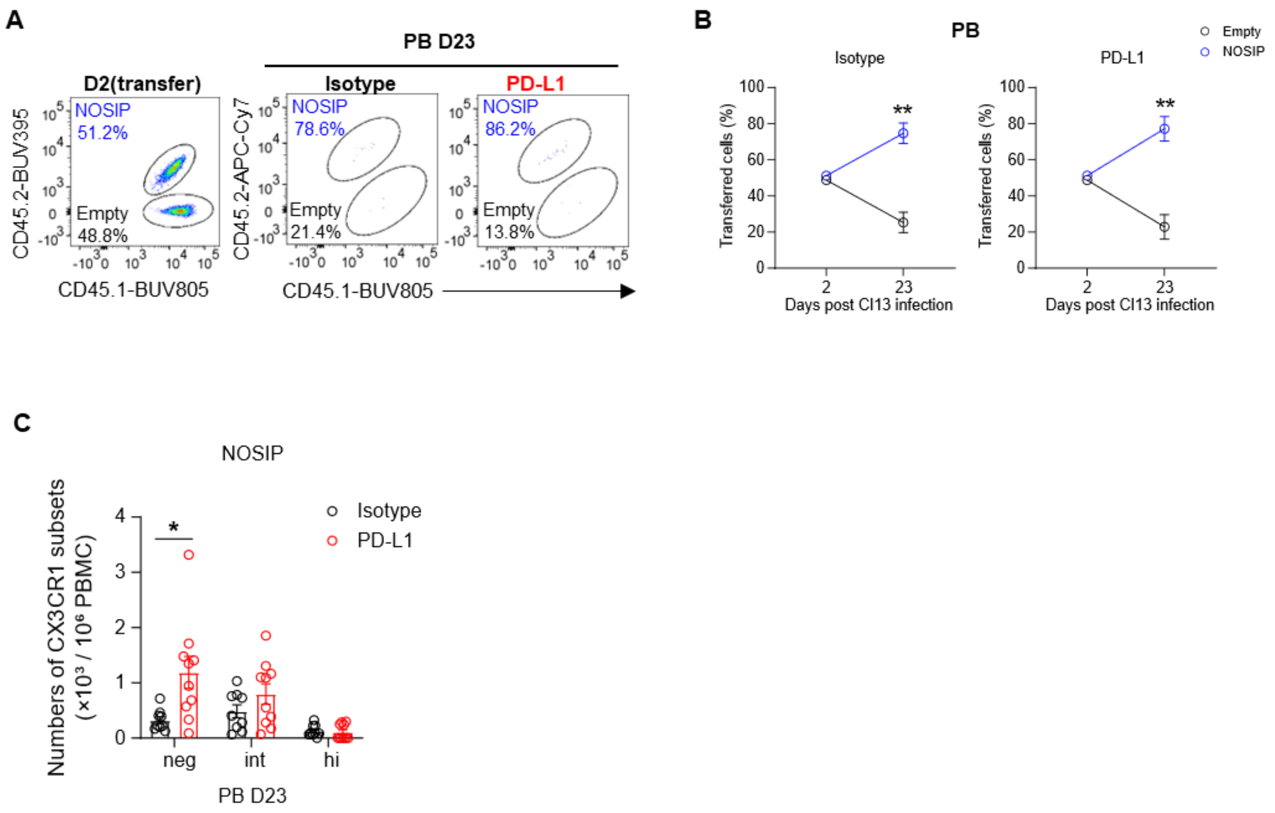

Supplementary Figure 9. NOSIP-overexpressing CD8^{+} T cells show enhanced persistence in response to PD-L1 blockade

(A, B) Representative flow plots (A) and quantification (B) showing frequencies of P14 cells in PB at day 2 (transfer) and day 23 post-infection, presented separately for isotype- and anti–PD-L1–treated groups. **(C)** Quantification of CX3CR1^{neg}, CX3CR1^{int}, and CX3CR1^{hi} subsets among NOSIP-transduced P14 cells in PB at day 23 post-infection, normalized per 10^6 PBMC. Data are pooled from two independent experiments. Bars represent mean ± s.e.m. Statistical significance was determined using a paired two-tailed Student's t-test for (B), unpaired two-tailed t-tests with Welch's correction for (C). $^*P < 0.05$, $^{**}P < 0.01$.

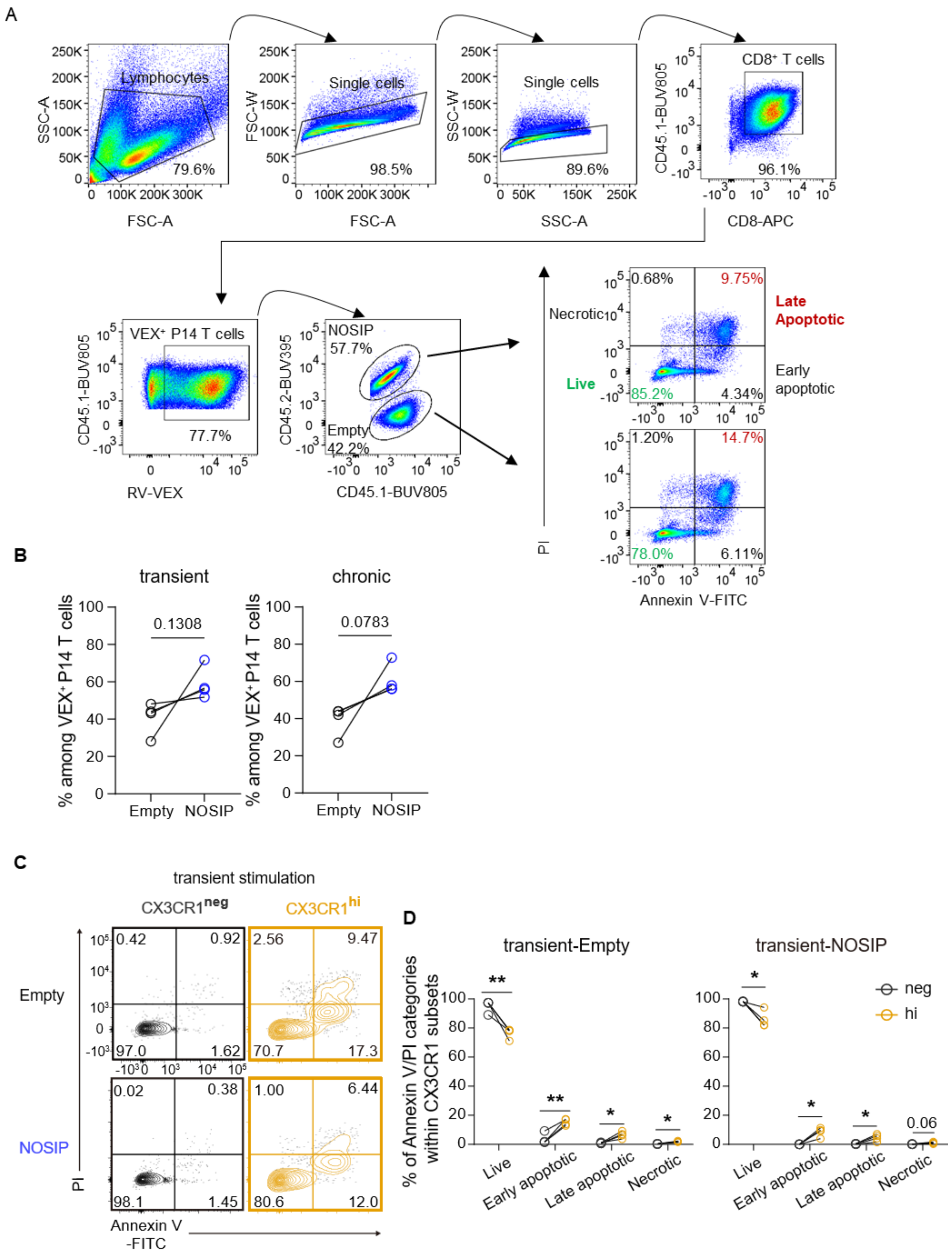
A
Lymphocytes
79.6%
Single cells
98.5%
Single cells
89.6%
CD8⁺ T cells
96.1%
SSC-A
FSC-A
FSC-W
SSC-W
CD45.1-BUV805
CD8-APC
VEX⁺ P14 T cells
77.7%
RV-VEX
NOSIP
57.7%
Empty
42.2%
CD45.2-BUV395
CD45.1-BUV805
Necrotic
Late Apoptotic
Live
Early apoptotic
0.68%
9.75%
85.2%
4.34%
1.20%
14.7%
78.0%
6.11%
PI
Annexin V-FITC
B
transient
chronic
0.1308
0.0783
% among VEX⁺ P14 T cells
Empty
NOSIP
C
transient stimulation
CX3CR1neg
CX3CR1hi
Empty
NOSIP
0.42
0.92
97.0
1.62
2.56
9.47
70.7
17.3
0.02
0.38
98.1
1.45
1.00
6.44
80.6
12.0
PI
Annexin V -FITC
D
transient-Empty
transient-NOSIP
% of Annexin V/PI categories within CX3CR1 subsets
neg
hi
Live
Early apoptotic
Late apoptotic
Necrotic
0.06

Supplementary Figure 10. ***In vitro*** **apoptosis analysis by Annexin V/PI**

(A) Gating strategy related to Figure 5 to distinguish empty- and NOSIP-transduced populations based on CD45.1/CD45.2 expression. Apoptosis was assessed by annexin V and PI staining. Representative plots showing live (Annexin$^-$ PI$^-$), early apoptotic (Annexin$^+$ PI$^-$), late apoptotic (Annexin$^+$ PI$^+$), and necrotic (Annexin$^-$ PI$^+$) VEX$^+$ P14 cells. **(B)** Frequency of VEX$^+$ P14$^+$ cells in empty and NOSIP groups under transient and chronic stimulation, corresponding to Figure 5 (B). **(C)** Representative plots showing annexin V and PI staining of CX3CR1^{neg} and CX3CR1^{hi} subsets in empty or NOSIP groups under transient stimulation. **(D)** Annexin V/PI categories (%) (live, early apoptotic, late apoptotic, necrotic) within CX3CR1^{neg} and CX3CR1^{hi} subsets in Empty or NOSIP groups under transient stimulation. The data represent 4 biological replicates, and each experiment was performed with technical duplicates. Statistical significance was determined using a paired two-tailed Student's t-test. Trends ($0.05 < P < 0.1$) are indicated where relevant.

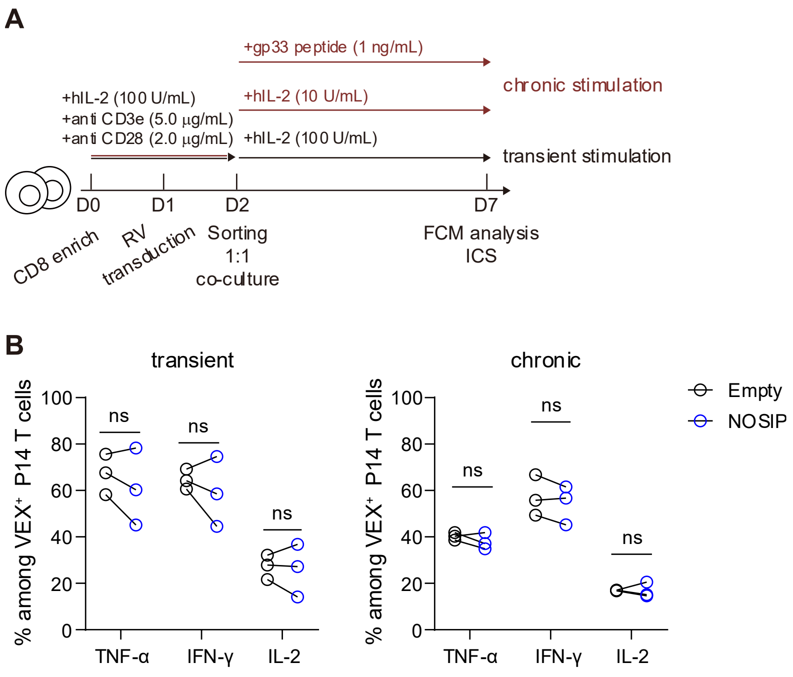

Supplementary Figure 11. NOSIP-overexpressing CD8⁺ T cells remain functionally competent *in vitro*

(A) Experimental timeline of the co-culture assay for intracellular cytokine staining. VEX^+ P14 cells were sorted on day 2 and analyzed on day 7. **(B)** Intracellular staining of TNF-α, IFN-γ, and IL-2 in VEX^+ P14 cells under transient and chronic stimulation. The data represent 4 biological replicates, and each experiment was performed with technical duplicates. Statistical significance was determined using a paired two-tailed Student's t-test. ns, not significant.

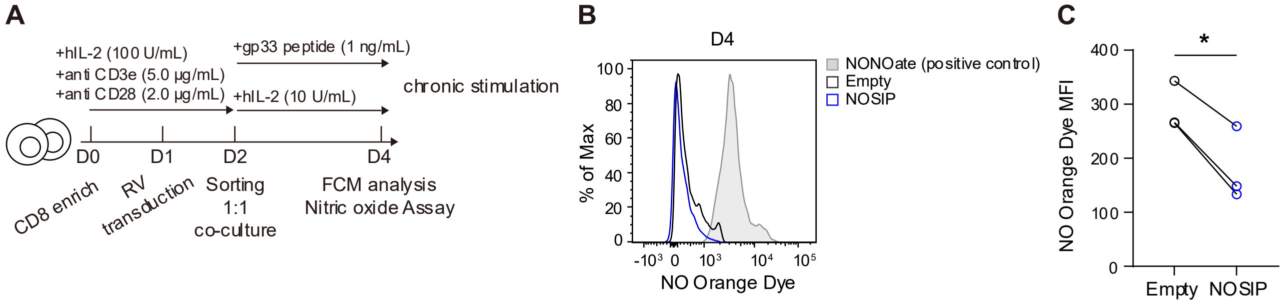

Supplementary Figure 12. Assessment of intracellular NO level under chronic stimulation *in vitro*

(A) Experimental scheme of the NO assay. VEX$^+$ empty and NOSIP-overexpressing P14 cells were sorted on day 2, co-cultured at a 1:1 ratio under chronic stimulation, and analyzed on day 4. **(B)** Representative histogram of NO Orange Dye staining. NONOate-treated cells were used as a positive control. **(C)** Quantification of NO Orange Dye MFI.
